# Targeting of multiple tumor-associated antigens by individual T cell receptors during successful cancer immunotherapy

**DOI:** 10.1016/j.cell.2023.06.020

**Published:** 2023-07-24

**Authors:** Garry Dolton, Cristina Rius, Aaron Wall, Barbara Szomolay, Valentina Bianchi, Sarah A.E. Galloway, Md Samiul Hasan, Théo Morin, Marine E. Caillaud, Hannah L. Thomas, Sarah Theaker, Li Rong Tan, Anna Fuller, Katie Topley, Mateusz Legut, Meriem Attaf, Jade R. Hopkins, Enas Behiry, Joanna Zabkiewicz, Caroline Alvares, Angharad Lloyd, Amber Rogers, Peter Henley, Christopher Fegan, Oliver Ottmann, Stephen Man, Michael D. Crowther, Marco Donia, Inge Marie Svane, David K. Cole, Paul E. Brown, Pierre Rizkallah, Andrew K. Sewell

**Affiliations:** 1 Division of Infection and Immunity, Cardiff University School of Medicine, Cardiff, Wales CF14 4XN, UK; 2 Systems Immunology Research Institute, Cardiff, Wales CF14 4XN, UK; 3 Division of Cancer and Genetics, Cardiff University School of Medicine, Cardiff, Wales CF14 4XN, UK; 4 National Center for Cancer Immune Therapy, Department of Oncology, Copenhagen University Hospital, Herlev, Denmark; 5 The Zeeman Institute, University of Warwick, Coventry CV4 7AL, UK

## Abstract

The T cells of the immune system can target tumors and clear solid cancers following tumor-infiltrating lymphocyte (TIL) therapy. We used combinatorial peptide libraries and a proteomic database to reveal the antigen specificities of persistent cancer-specific T cell receptors (TCRs) following successful TIL therapy for stage IV malignant melanoma. Remarkably, individual TCRs could target multiple different tumor types via the HLA A*02:01-restricted epitopes EAAGIGILTV, LLLGIGILVL, and NLSALGIFST from Melan A, BST2, and IMP2, respectively. Atomic structures of a TCR bound to all three antigens revealed the importance of the shared x-x-x-A/G-I/L-G-I-x-x-x recognition motif. Multi-epitope targeting allows individual T cells to attack cancer in several ways simultaneously. Such “multipronged” T cells exhibited superior recognition of cancer cells compared with conventional T cell recognition of individual epitopes, making them attractive candidates for the development of future immunotherapies.

## Graphical abstract

**Graphical abstract:**
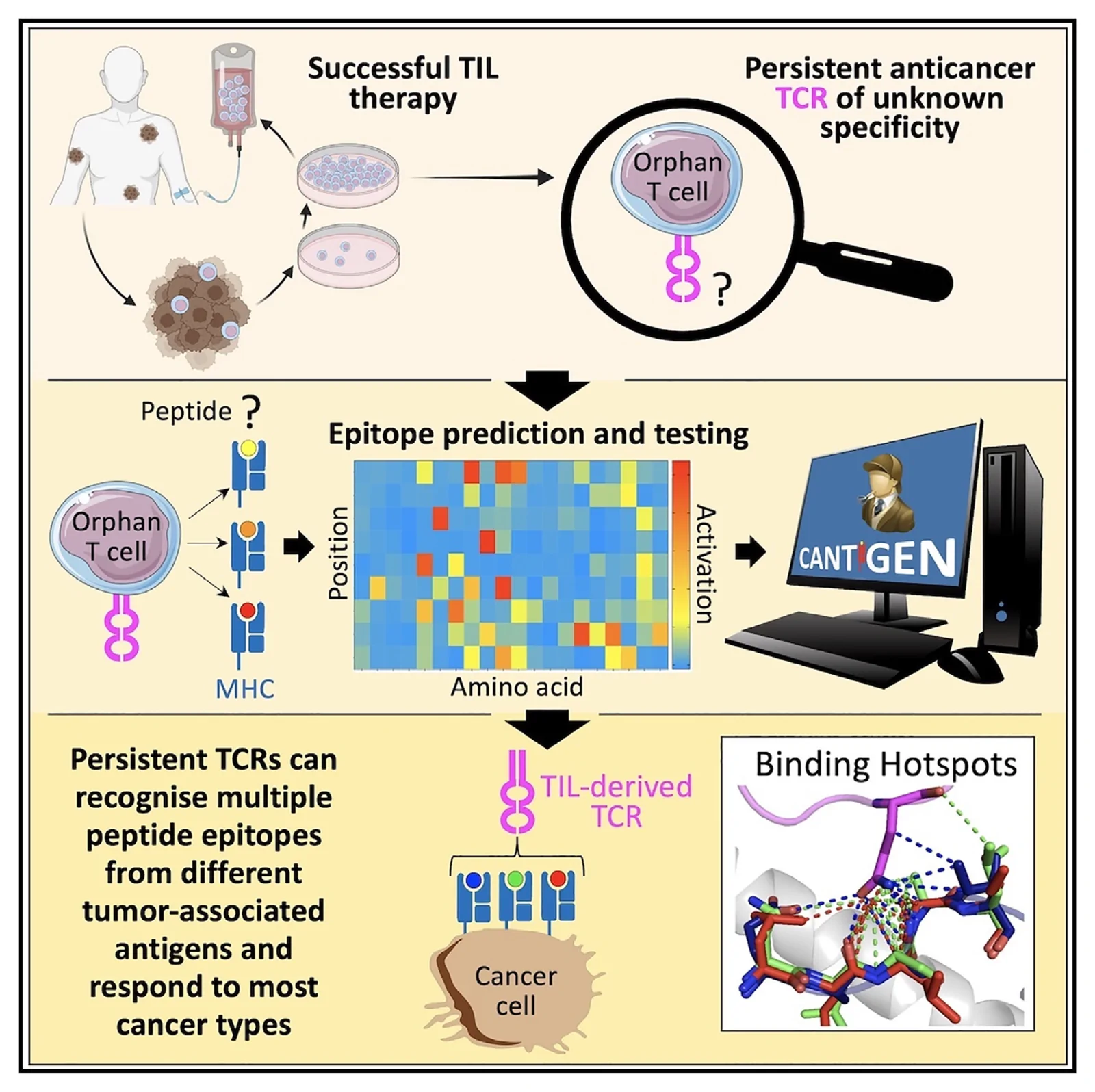

## Introduction

The human leukocyte antigen class I (HLA-I) presentation pathway displays the cellular proteome at the cell surface in the form of short peptides. These peptide-HLA molecules are interrogated by T cell receptors (TCRs) on the surface of cytotoxic T lymphocytes (CTLs).^1^ This system allows CTLs to detect and destroy cells containing anomalies in protein expression caused by intracellular pathogens or cellular transformation. The ability of CTLs to distinguish the proteome of healthy cells from that of cancer cells has been exploited using checkpoint blockade^2^ and tumor-infiltrating lymphocyte (TIL) therapy^3^ to result in the biggest development in cancer therapy for over 50 years. The process of T cell selection in the thymus^4^ determines that T cells with TCRs that have capacity to bind to peptides from tumor-associated antigens (TAAs) bind relatively weakly compared with those that recognize pathogen-derived (non-self) peptides.^5^ As TCR affinity governs the functional profile of T cells, natural cancer-specific T cells are known to respond sub-optimally to natural levels of peptide on the cancer cell surface.^6^ Nevertheless, the induction of complete remission of solid tumors following checkpoint blockade and TIL therapies suggests that natural TCR interactions with peptide-HLA can irradicate cancer.

The antigens and epitopes targeted by T cells during “successful” cancer immunotherapy remain largely unknown. We studied autologous tumor-specific T cells in the TIL infusion product used to induce complete durable remission in HLA A*02:01^*+*^ stage IV malignant melanoma patient MM909.24.^7^ Positional scanning combinatorial peptide library (PS-CPL) screening^8^ was used in conjunction with a proteomic database, search algorithms,^9,10^ and confirmatory recognition assays to develop a platform for discovery of the ligands recognized by cancer-reactive T cells of unknown specificity (so-called “orphan” T cells). This platform, named CANTiGEN, successfully predicted the antigen recognized by eight different cancer-specific TCRs and identified that, in addition to the EAAGIGILTV peptide derived from Melan A, the dominant Melan-A-reactive T cell clone in the TIL used to successfully treat patient MM909.24, MEL8, also recognized two new cancer-associated epitopes: LLLGIGILVL from tetherin, also known as CD317 and bone marrow stromal antigen 2 (BST2)^11^ and NLSALGIFST from insulin-like growth factor 2 mRNA-binding protein 2 (IMP2)^12^ in the context of HLA A*02:01. The MEL8 T cell clone stained with HLA A*02:01 tetramers made with all three epitopes. Staining of the TIL product used to treat patient MM909.24 with tetramers of the three antigens indicated that the patient was infused with over 9.7 × 10^9^, 4.7 × 10^9^, and 9.6 × 10^9^ Melan A-, BST2-, and IMP2-reactive T cells, respectively, and that several other T cell clonotypes within the TIL infusion product could respond to all three antigens while the well described DMF4 TCR that was previously used in TCR-T adoptive cell therapy^13^ only responded to Melan A. We adopted the term “multipronged” to describe T cells that could simultaneously target cancer via multiple TAAs. Importantly, recognition via each TAA was additive and resulted in superior recognition of cancer cells by multipronged T cells compared with conventional T cells that recognized cancer cells via a single TAA. Our discovery that single successful TCRs can recognize many cancers via several different HLA-presented epitopes makes such TCRs attractive propositions for exploitation during immunotherapy.

## Results

### Patient MM909.24 TIL infusion product responds to widely shared HLA A*02:01-restricted antigens

**Figure 1:**
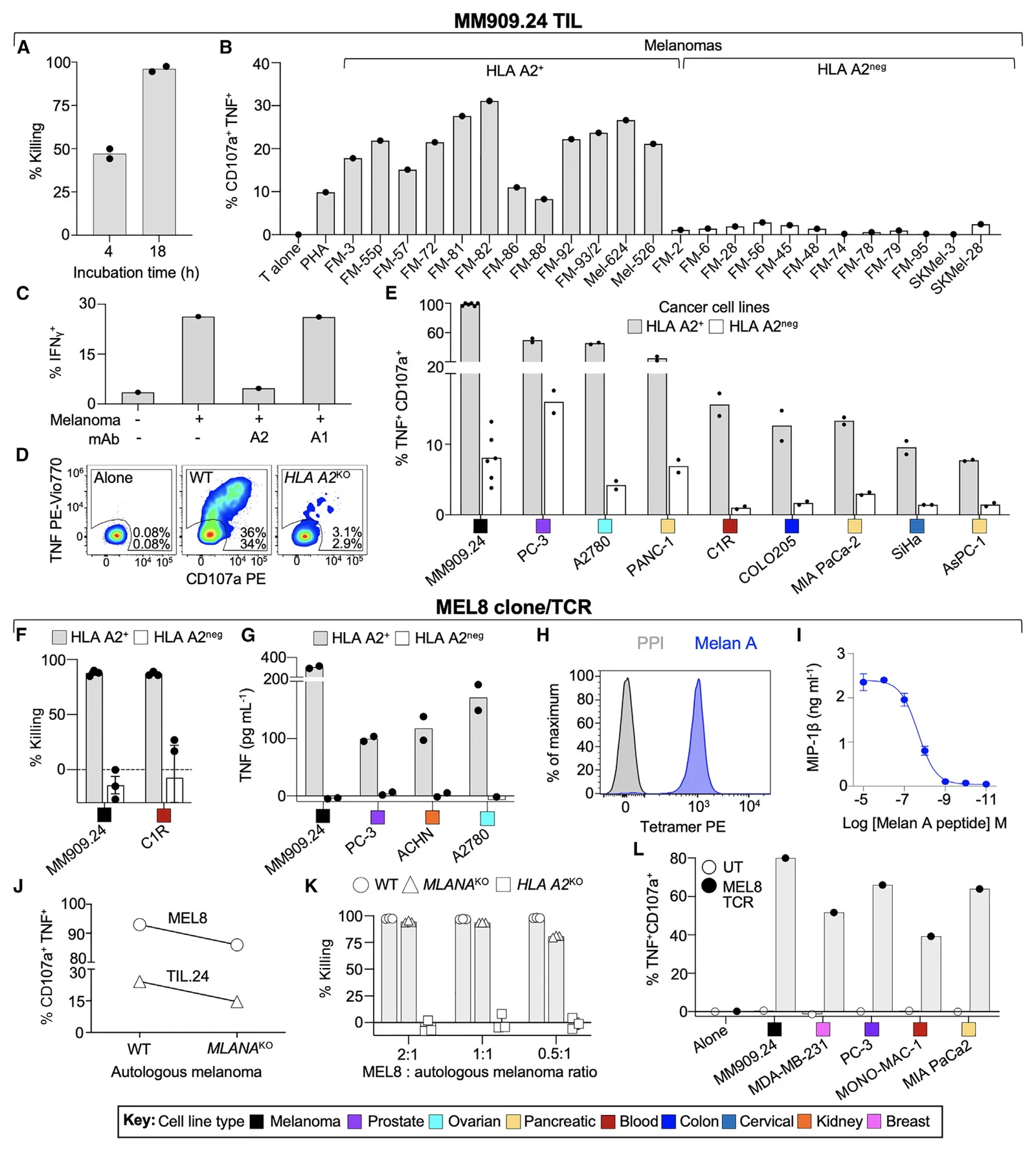
MM909.24 TIL and derived MEL8 T cell clone recognize multiple cancer cell types via shared HLA A*02:01-restricted antigens (A) Cytotoxicity assay (Chromium^51^) of TIL (passage-2) against autologous melanoma. (B) T107 assay (4 h) with TIL (passage-2) and HLA A*02:01^*+*^ or HLA A*02:01^neg^ melanoma cell lines. (C) Reactivity (intracellular IFNγ after 4 h) of TIL with autologous melanoma ± HLA-A*02 or -A*01 antibodies. (D) T107 assay (4 h) of TIL with wild-type (WT) or HLA A*02:01 knockout (KO) autologous melanoma cell line. (E) T107 assay (5 h) of TIL (passage-2) with isogenic cancer cell lines ± HLA A*02:01 (cell line type in key). Performed over three assays with normalization to WT autologous melanoma cells. (F) Killing (48 h) of MEL8 clone versus autologous melanoma and C1R cells ± HLA A*02:01. Error bars depict SEM of triplicate conditions. (G) Overnight MEL8 activation (TNF ELISA) with cancer cell lines ± HLA A*02:01. (H) Staining of MEL8 with Melan A_26–35_ (EAAGIGILTV) or irrelevant preproinsulin_15–24_ (PPI) tetramers. (I) Overnight MEL8 activation (MIP-1β ELISA) with Melan A peptide. (J) T107 assay (4 h) of MEL8 and TIL (passage-3) with WT or *MLANA* (Melan A) knockout autologous melanoma cells. (K) Killing assay (24 h) with MEL8 and WT, *MLANA* knockout and HLA A*02:01 knockout autologous melanoma cells. Error bars depict SEM of triplicate conditions. (L) CD8 T cells ± MEL8 TCR against cancer cell lines ± HLA A*02:01. Percentage TNF^+^/CD107a^+^ of CD8^+^/rCD2(TCR co-marker)^+^ cells, minus background with HLA A*02:01^*neg*^ cancer cell lines is displayed. Genetic modifications in Figure S1.

Stage IV melanoma patient MM909.24 underwent therapy with 1.1 × 10^11^ autologous TIL and remains cancer free a decade after treatment.^14^ MM909.24 expresses HLA A*02:01, the most common HLA allele across the global population.^15^ We have previously demonstrated that up to 50% of the TIL infusion product used to treat this patient responded to the patient autologous melanoma line.^16^ These TIL exhibit robust killing of the autologous melanoma cell line and respond to other HLA A*02:01^*+*^ melanoma lines (Figures 1A and 1B) suggesting that the majority of the response to the autologous tumor line might be via shared HLA A*02:01-restricted epitopes rather than through patient-specific, mutation-generated neoantigens. Anti-HLA A*02 antibody, but not irrelevant anti-HLA A*01 antibody, blocked almost all the response to the autologous tumor line (Figure 1C), and ablation of the HLA A*02:01 gene (Figure S1) reduced the response to the autologous melanoma line by over 90% (Figure 1D) indicating that the majority of the response to the autologous tumor line acted through this HLA. Surprisingly, MM909.24 TIL responded to many cancer cell lines of differing origin through HLA A*02:01 (Figures 1E, S1A, and S1B for HLA A*02:01 expression/ablation). HLA A*02:01-restricted TCRs that recognize shared antigens expressed by a wide variety of cancer types were of special interest, so we tested whether T cell clone MEL8, grown from MM909.24 TIL infusion product by limiting dilution, could respond to other cancer types in addition to melanoma. MEL8 responded to a variety of HLA A*02:01^+^ cancer cells (Figures 1F, 1G, S1A, and S1B). Recognition of non-melanoma cancer cell lines by this T cell clone was particularly surprising as it recognizes the well-studied 10-mer EAAGIGILTV epitope (Melan A_10_) (peptide abbreviations and summary; Table S1) from the Melan A protein (Figures 1H and 1I), which is overexpressed by 90% of cutaneous melanomas.^16,17^ Melan A is not expressed by other cancer cell types (Figures S1C and S1D), suggesting that MEL8 might respond to a broadly expressed TAA through HLA A*02:01 in addition to recognizing the Melan A_10_ epitope. To confirm that MEL8 recognized a TAA in addition to Melan A, we used CRISPR-Cas9 to knockout (KO) the *MLANA* gene that encodes this protein in the MM909.24 melanoma line (Figures S1C and S1D). CRISPR-Cas9 KO of Melan A in the autologous melanoma line reduced activation of MM909.24 TIL by ~10% (Figure 1J). Killing of the Melan A KO line by MEL8 was minimally affected (Figure 1K), suggesting that the MEL8 T cell clone recognized the patient tumor via more than one HLA A*02:01-restricted epitope. Clonotyping confirmed that MEL8 was clonal and expressed single TCRα and TCRβ chains (Figure S2) affirming that the reactivity seen against multiple cancer types was through a single TCR. Indeed, the MEL8 TCR conferred reactivity toward autologous melanoma and a variety of cancer cell types (breast, prostate, leukemia, and pancreatic) when introduced to primary CD8 T cells from a healthy donor (Figure 1L). We next set out to discover the second epitope that was recognized by the MEL8 T cell clone and that appeared to be expressed by multiple cancer types. This required that we develop robust methodology for the discovery of the ligands recognized by orphan cancer-specific T cells.

### A pipeline for discovery of cancer-associated T cell epitopes

We have previously used vast, yet manageable, combinatorial peptide libraries (e.g., 9.36 × 10^11^ different peptides for a 10-mer library) to generate data that allow successful prediction of strong agonist sequences using search algorithms.^8–10^ We adapted our pipeline (Figures S3A–S3C) to the discovery of peptides recognized by cancer-specific T cells. PS-CPL data from five different T cell clones (Figures S3D and S3E) were used to search a human proteome database for potential agonist ligands. All searches put the real peptide within the top 500 peptides from the human proteome (Figure S3F), but this number was deemed too large to allow inexpensive, rapid functional verification. To improve prediction results, we built a human cancer proteome database of full-length tumor-associated protein sequences, and described peptides derived thereof (CANTiGEN; https://cantigen.cf.ac.uk//launch.php, see graphical abstract for the CANTiGEN pipeline and Figures S3A–S3C). In searches of the CANTiGEN database and algorithm prediction the real epitope was within the top 12 predicted peptides for four of the clones, and at position 39 for the fifth clone (Figure S3F). Having established the versatility of our approach for identification of known cancer epitopes we next applied it to orphan, autologous cancer-specific T cell clones isolated from the TIL used to successfully treat stage IV melanoma patients (Figure S4). CANTiGEN correctly identified that orphan HLA A*02:01-restricted T cell clone VB8 from patient MM909.24 TIL recognized the 9-mer AAGIGILTV peptide (Melan A_9_; Table S1) (Figures S4A–S4F). We further applied the technology to orphan T cell clone GD.2.11, which was isolated from the TIL used to successfully treat HLA A*02:01-negative patient MM909.11.^7,18^ CANTiGEN correctly predicted that the sequence RLSNRLLLR from a cancer/testis tumor antigen gene (TAG) as the likely antigen in the context of HLA A*03:01 (Figures S4G–S4M). T cell clone GD.2.11 responded to the TAG peptide (Figure S4K), stained with a HLA A*03:01-TAG tetramer (Figure S4L), and responded to HLA A*03:01^+^ melanoma cell lines but not those that that did not express this allele (Figure S4M). The TILs from MM909.11 and a second HLA A*03:01^+^ complete remission melanoma patient (MM909.15) stained 0.67% and 0.05%, respectively, with TAG tetramer (Figure S4N). Based on the percentage of tetramer^+^ cells and the reactivity of these TILs toward autologous melanoma (MM909.11, 3.8% and MM909.15, 4.5%, data not shown), the TAG T cells make up 17.5% (MM909.11) and 1.1% (MM909.15) of the anti-melanoma response seen for the TILs used to treat these patients. TAG-peptide-specific T cells have been detected in the blood of patient MM909.11 post TIL infusion^18^ suggesting that a large number of activated T cells that bind to the TAG peptide is well tolerated. We conclude that the CANTiGEN peptide searching tool was efficient for finding relevant peptides from shared TAA regardless of the restricting HLA class I molecule.

### The T cell clone MEL8 responds to three different TAA

**Figure 2:**
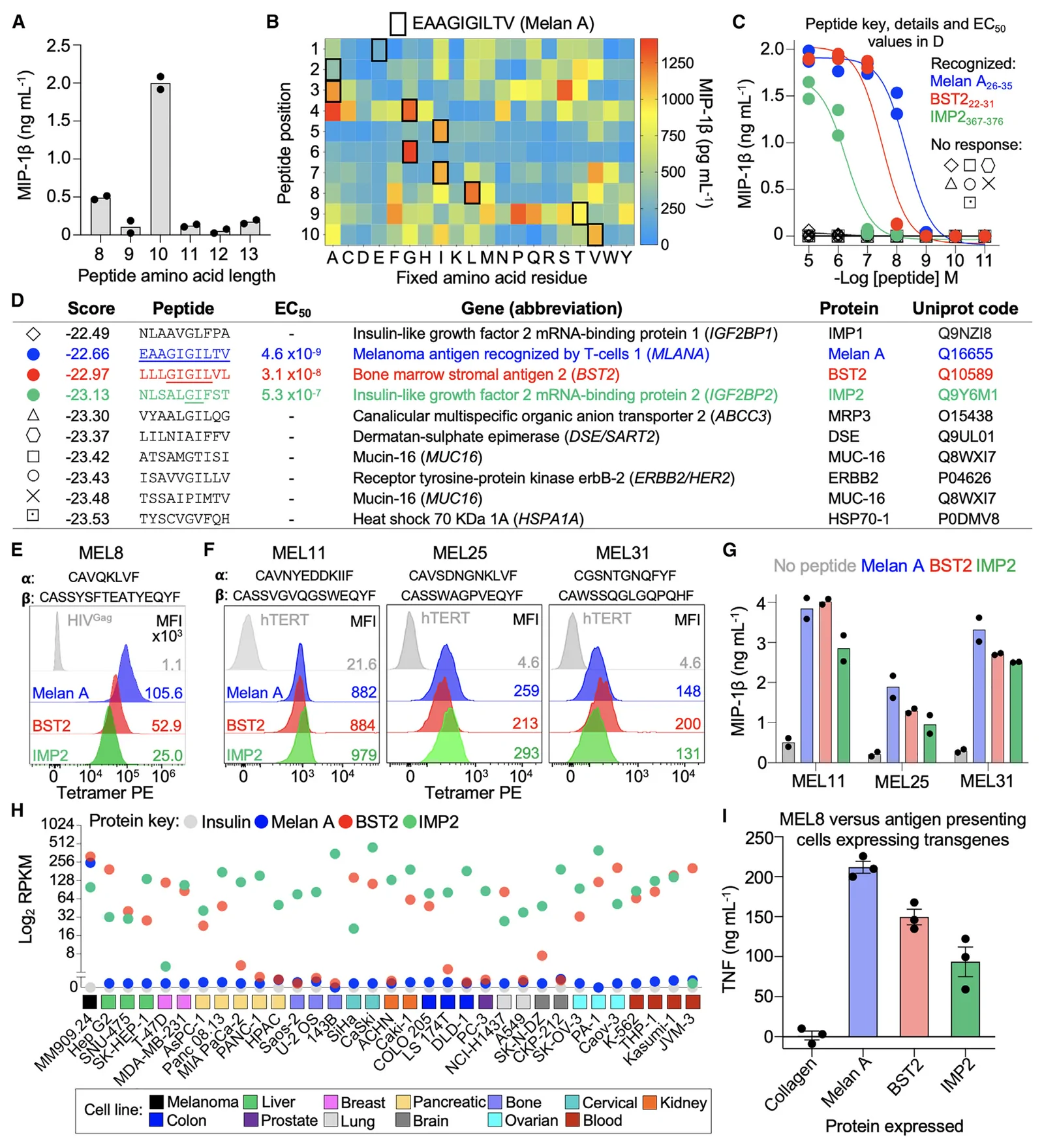
The MEL8 T cell clone recognizes multiple unique peptides from different TAAs (A) Peptide length preference of MEL8 T cell clone measured by MIP-1β ELISA. (B) Heatmap of MEL8 activation (MIP-1β ELISA) from a positional scanning combinatorial peptide library (PS-CPL) screen. (C) Activation (MIP-1β ELISA) of MEL8 with the top 10 peptides identified using the PS-CPL data and CANTiGEN. Peptide details and EC_50_ values in (D). (D) Peptides tested with MEL8 in (C). Peptides are ranked/scored for likelihood of recognition by MEL8. Amino acids common to Melan A, BST2, and IMP2 peptides are underlined. (E) Staining of MEL8 with Melan A, BST2, IMP2, and irrelevant (HIV-Gag_77–85_)^20^ tetramers. Complementarity determining regions 3 and mean fluorescence intensity (MFI) are displayed. (F) Clones with unique TCRs from the TIL of patient MM909.24 stained with tetramers as in (E). Irrelevant tetramer human telomerase reverse transcriptase_540–548_ (hTERT).^21^ (G) Overnight peptide (10^−6^ M) activation (MIP-1β ELISA) of clones in (F). (H) RNA-seq data of Melan A, BST2, and IMP2 in MM909.24 melanoma and cancer cell lines of various tissue origins. Insulin plotted as a negative control. RNA-seq data for tumor and matched healthy tissue in Figure S5. (I) Overnight activation (TNF ELISA) of MEL8 with MOLT3 cell line + HLA A*02:01 transduced with genes for Melan A, BST2, or IMP2. Collagen gene as a negative control.

We put the MEL8 T cell clone through our CANTiGEN screening process. A sizing scan^19^ showed that the MEL8 T cell clone preferred 10-mer peptides (Figure 2A) in accordance with it recognizing the Melan A_10_ peptide. 10-mer PS-CPL data generated using MEL8 (Figure 2B) were used for CANTiGEN screening. Remarkably, the MEL8 T cell clone recognized three of the top ten predicted and tested peptides (Figures 2C and 2D): Melan A_10_, LLLGIGILVL from BST2 (BST2_10_), and NLSALGIFST from IMP2 (IMP2_10_; Table S1). Additionally, the MEL8 T cell clone stained with HLA A*02:01 tetramers presenting Melan A_10_, BST2_10_, and IMP2_10_ (Figure 2E). Whereas CANTiGEN ranked the former peptides at positions 2, 3, and 4, respectively, a self-database screen using the same MEL8 PS-CPL ranked them at 68, 135, and 210, further highlighting the efficiency of the CANTiGEN database to find cancer-associated peptides that activate orphan T cell clones. All three agonist peptides have common residues at positions 6 (Gly) and 7 (Ile), with the Melan A_10_ and BST2_10_ peptides sharing five amino acids at positions 4–8 (Gly-Ile-Gly-Ile-Leu) (Figure 2D). Having established that persistent T cell clonotype MEL8 could exhibit this remarkable behavior, we next went back to examine three other T cell clonotypes MEL11, MEL25, and MEL31 isolated from the TIL used to successfully treat patient MM909.24. All three T cell clones stained with all three tetramers and responded to each peptide in activation assays (Figures 2F and 2G). The BST2 and IMP2 genes are expressed by the autologous melanoma, ubiquitously by most cancers (Figure 2H) and overexpressed relative to normal tissue (Figure S5), making potential HLA A*02:01-restricted peptide epitopes from these TAA particularly interesting. MEL8 recognized HLA A*02:01-expressing target cell lines co-transduced with genes encoding Melan A, BST2, or IMP2 but not one expressing collagen, indicating that the corresponding peptides are processed and presented at the cell surface (Figure 2I).

### Individual TCRs can bind to peptides from three different TAA

**Figure 3:**
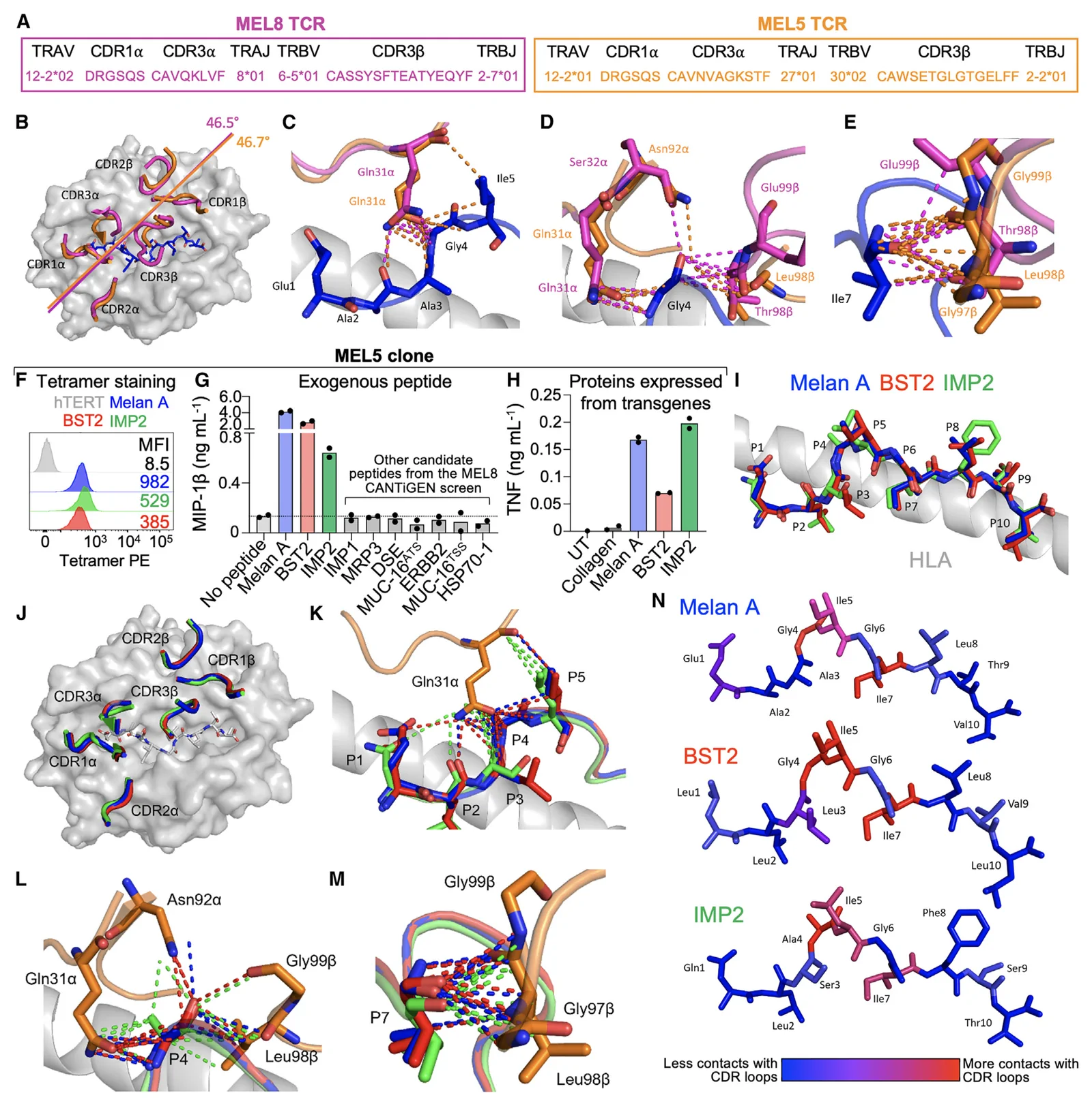
Recognition of three different tumor-associated epitopes by a single TCR (A) MEL8 (magenta) and MEL5 (orange, PDB: 4QOK) TCR genes andcomplementarity determining regions (CDRs) 1/3 sequences: TCR color used throughout unless stated. (B) Comparison of MEL8 and MEL5 TCR crossing angles and CDR loops in relation to HLA A*02:01 (gray) and Melan A_26–35_ (EAAGIGILTV) peptide (blue). (C) Close up of MEL8 and MEL5 TCR residue Gln31α interacting with the Melan A peptide (blue). (D and E) Comparison between MEL8 and MEL5 TCR residues interacting with Gly4 and Ile7 of the Melan A peptide (blue). (F) Tetramer staining of MEL5 T cell clone with indicated tetramers. MFI, mean fluorescence intensity. (G) Overnight activation (MIP-1β ELISA) of MEL5 with the top 10 peptides (10^−6^ M) identified by the MEL8 CANTiGEN screen. (H) MEL5 recognition (TNF ELISA) of MOLT3 cell line + HLA A*02:01, expressing genes for Melan A, BST2, or IMP2. Untransduced (UT) and Collagen gene used as negative controls. (I) Overlay of Melan A (blue), BST2 (red), and IMP2 (green) peptides from MEL5-pMHC co-structures. MHC alpha helix (gray) shown as cartoon for orientation. (J) Top-down view of the MEL5 TCR with CDR3 loops color coded according to the peptide: Melan A (blue), BST2 (red), or IMP2 (green). Representative peptide shown in white. (K) Close up of MEL5 Gln31α interacting with the Melan A (blue), BST2 (red), and IMP2 (green) peptides. (L and M) MEL5 residues interactions with P4 and P7 of Melan A (blue), BST2 (red), and IMP2 (green), with bonds colored according to the peptide. (N) Heatmap of MEL5 TCR CDR loop interactions with Melan A, BST2, and IMP2 peptides.

We generated crystals of the MEL8 TCR in complex with HLA A*02:01-Melan A_10_ that diffracted to 3.24 Å (Figure S6A; Table S3). The structure of MEL8:HLA A*02:01-Melan A_10_ showed upward protrusion of Melan A_10_ peptide residues Glu1, Ile5, and Leu8 (Figure S6B). The MEL8 complementarity determining region (CDR) loop distribution shows that the CDR1α and CDR3β loops are in close proximity to the Melan A_10_ peptide (Figure S6C). Furthermore, MEL8 TCR residue Gln31α occupies a “pocket” formed by peptide residues P1–P5 (Figure S6D). Closer examination of the bonds between the Melan A_10_ peptide and the MEL8 TCR show many contacts (54% of van der Waals and 66% of hydrogen bonds) involve peptide residues Gly4 and Ile7. In contrast, peptide residues Glu1, Ile5, and Leu8 contribute less interactions (24% of van der Waals and no hydrogen bonds), despite their protrusion toward the MEL8 TCR (Figures S6E–S6G; Table S4). Crystals of MEL8 TCR in complex with the BST2_10_ and IMP2_10_ peptides yielded poor X-ray diffraction. Analysis of the MEL8:HLA A*02:01-Melan A_10_ structure prompted comparison with the previously published MEL5:HLA A*02:01-Melan A_10_ structure.^22^ The MEL5 T cell was originally selected for study from over 40 different T cell clones grown out from healthy donors using the heteroclitic Melan A epitope ELAGIGILTV (Melan A_A2L_) as MEL5 showed the best killing of HLA A*02:01^*+*^ melanoma cell lines Mel624 and Mel526^23^ of all the clones so was taken forward for further analyses.^24^ A comparison between the MEL8:HLA A*02:01-Melan A_10_ and MEL5:HLA A*02:01-Melan A_10_ structures show conserved CDR1α conformations (both TRAV12-2*02 and 12-2*01 have the same CDR1), with TCR residue Gln31 occupying a P1–P5 pocket. While the differing CDR3 loop compositions results in differing CDR3 conformations, the focus on the Gly4 and Ile7 hotspots remains consistent between both the MEL8 and MEL5 TCRs (Figures 3A–3E). This similarity led us to examine whether the MEL5 T cell could also recognize the BST2_10_ and IMP2_10_ epitopes like MEL8. The MEL5 T cell clone stained well with all three tetramers (Figure 3F) and recognized these peptides in activation assays (Figure 3G). MEL5 did not recognize the other seven peptides from the top ten candidates identified from the PS-CPL and CANTiGEN screening of MEL8 (Figure 3G); consistent with the MEL8 activation data (Figure 2C). MEL5 also recognized HLA A*02:01^*+*^ targets expressing Melan A, BST2, or IMP2 proteins (Figure 3H), again proving that the peptides are genuine epitopes. Comparison of the MEL8 and MEL5 TCRs transduced in to CD8^+^ Jurkat cells showed similar staining with BST2_10_ and IMP2_10_ tetramers (Figure S7A). CD8 T cells from the same donor transduced with the MEL8 or MEL5 TCRs gave the same hierarchy of activation for each of the peptides, with Melan A_10_ being seen the best, followed by BST2_10_ then IMP2_10_ (Figure S7B). In the absence of good diffraction data for the MEL8 TCR in complex with the BST2 and IMP2 peptides we reasoned that the MEL5 TCR would act as a good surrogate for the structural component of this study as we have previously generated good crystals of the MEL5 TCR in complex with HLA A*02:01-Melan A_A2L_,^24^ HLA A*02:01-Melan A_10_,^22^ and HLA A*02:01-Melan A_9_.^25^ We generated crystals of the MEL5 TCR in complex with the BST2_10_ and IMP2_10_ epitopes that diffracted to 2.1 and 2.55 Å, respectively (Figure S6A; Table S3). Overlay of the Melan A_10_, BST2_10_, and IMP2_10_ epitopes within the complex structures showed that they adopted a similar conformation (Figure 3I) allowing a conserved TCR docking mechanism despite the different peptide sequences (Figure 3J). The MEL5 TCR focuses on the shared x-x-x-A/G-L/I-G-I-x-x-x motif between the Melan A_10_, BST2_10_, and IMP2_10_ epitopes demonstrating that MEL5 TCR crossreactivity is driven by molecular mimicry between all three epitopes (Figures 3K–3N; Table S3). We conclude that individual T cells can target cancer via epitopes from three different TAA. This type of multipronged recognition of target cells has never even been proposed before. While it was not possible to acquire the 3D structure of the unbound HLA A*02:01-BST2_10_ monomer, the 3D structure of the unbound HLA A*02:01-IMP2_10_ monomer successfully diffracted to 2.5 Å (Figures S6A and S6H; Table S4). Comparison between HLA A*02:01-IMP2_10_ indicates no conformation shifts occur within the peptide upon interaction with MEL5.

Homology modeling of the MEL8 TCR in complex with the BST2_10_ and IMP2_10_ epitopes suggests that molecular mimicry is responsible for MEL8 crossreactivity (Figures S6I–S6L). Further analysis of the modeled interactions between the MEL8 TCR and the cancer epitopes show that interactions involving the P4 and P7 hotspots account for 24% and 39% of the interactions involving the BST2_10_ and IMP2_10_ epitopes, respectively (Table S3). While the theoretical nature of the MEL8:HLA A*02:01-BST2_10_ and the MEL8:HLA A*02:01-IMP2_10_ structural models means these data carry certain caveats, they support the experimental data presented in Figure 2, further suggesting molecular mimicry as a mechanism for MEL8 crossreactivity.

### Recognition of each TAA-derived peptide by multipronged T cells is additive

**Figure 4:**
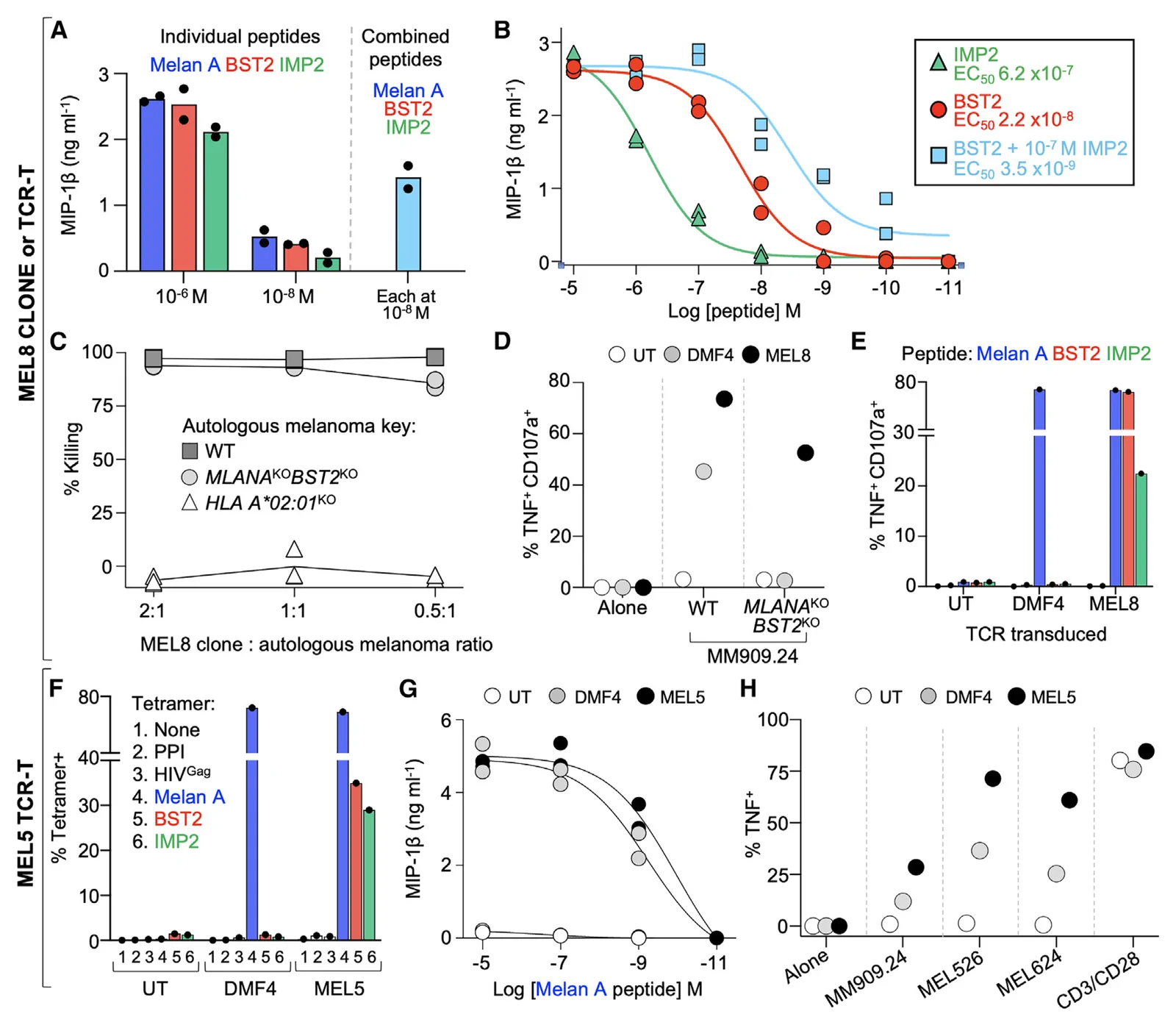
Recognition of Melan A, BST2, and IMP2 is additive, prevents cancer escape, and improves recognition of cancer cells (A) MEL8 clone activation (MIP-1β ELISA) with Melan A_26–35_ (EAAGIGILTV), BST2_22–31_ (LLLGIGILVL), and IMP2_367–376_ (NLSALGIFST) peptides, used alone or in combination. (B) MEL8 clone activation (MIP-1β ELISA) with BST2 (red) and IMP2 (green) peptides used individually, or in combination (turquoise). (C) MEL8 clone overnight killing of MM909.24 melanoma: WT, *MLANA/BST2* double knockout (KO), or HLA A*02:01 KO. (D and E) T107 assays (4 h) of MEL8 or DMF4 TCR-T cells. UT, untransduced. TNF^+^/CD107a^+^ of CD8^+^/rCD2(TCR co-marker)^+^ cells. (D) Tested against melanoma MM909.24 WT and *MLANA/BST2* KO cells. (E) Tested against multipronged peptides (10^−6^ M). (F) Staining of MEL5 or DMF4 TCR-T cells with multipronged and irrelevant (HIV-Gag_77–85_ or preproinsulin_15–24_)^20,27^ tetramers. Gated on CD8^+^/rCD2^+^. (G) Overnight activation (MIP-1β ELISA) of MEL5 or DMF4 TCR-T cells toward Melan A peptide. (H) Reactivity of MEL5 or DMF4 TCR-T cells toward HLA A*02:01^*+*^ melanoma cell lines. TNF^+^ of CD8^+^/rCD2^+^ cells. KO characterization in Figures S1C and S7G.

We next showed that recognition of Melan A_10_, BST2_10_, and IMP2_10_ epitopes is additive when they were supplied as exogenous peptides. Addition of all three peptides at 10 nM each enhanced activation by 2.7-, 3.5-, and 7-fold compared with individual Melan A_10_, BST2_10_, and IMP2_10_ peptides, respectively (Figure 4A). Addition of 100 nM of the weakest recognized peptide (IMP2_10_) shifted the response curve to the second weakest recognized peptide (BST2_10_) by almost a factor of 10 (EC_50_ 2.2 × 10^−8^ M for BST2_10_ alone compared with EC_50_ 3.5 × 10^−9^ M for BST2_10_ + 100 nM IMP2_10_; Figure 4B). While these data confirm that even a low amount of the weakest peptide agonist could augment recognition of other epitopes by MEL8 T cells, it may have little relevance in a physiological setting where protein antigens require processing and presentation at the tumor cell surface. To examine the contributions of each antigen in a more natural setting we turned to knocking out the antigens in the patient autologous melanoma line. This line failed to tolerate KO of the *IGF2BP2* (IMP2) gene, but we were able to KO the *MLANA* gene which encodes Melan A and *BST2* (Figure S7G) using CRISPR-Cas9 to leave only the weakest recognized of the three antigens IMP2. The MEL8 T cell line was still able to kill the autologous cancer cell line from which *MLANA* and *BST2* genes had been ablated (Figure 4C) confirming that even removal of the best two epitopes as endogenously supplied peptides did not prevent the patient’s own cancer line from being recognized. These results combine with those showing recognition of a wide range of cancer cell lines (Figure 1), even when some of these only express mRNA for BST2 or IMP2 (Figure 2H) to suggest that expression of any one of the three antigens at the level found in cancer cells is sufficient to render cells a target for MEL8 T cells. These results suggested that a multipronged TCR such as MEL8 would be better equipped at undermining tumor escape mechanisms that involve the loss of protein expression from cancer cells. With this in mind, we compared MEL8 to the clinically relevant Melan A_10_-specific DMF4 TCR, which mediated tumor regression as a TCR-T.^13^ Primary CD8 T cells from the same donor transduced with the MEL8 or DMF4 TCRs were tested against melanomas cells, which activated a greater proportion of the MEL8 transduced T cells compared with DMF4 (Figure 4D). The MEL8 TCR transduced T cells also out-performed DMF4 when tested against the Melan A and BST2 double KO melanomas (Figure 4D). The Melan A_10_ reactive DMF4 TCR transduced T cells did not respond to the BST2_10_ and IMP2_10_ peptides and is therefore monospecific, whereas the MEL8 TCR-T cells reacted to all three peptides (Figure 4E). Comparison of MEL5 to DMF4 TCR transduced T cells from the same donor showed similar advantages to being multipronged, as seen in the MEL8 TCR-T experiments. MEL5 transduced T cells stained with Melan A_10_, BST2_10_, and IMP2_10_ tetramers, whereas T cell expressing the DMF4 TCR only stained with the Melan A_10_ tetramer (Figure 4F). In titration assays with the Melan A_10_ peptide, MEL5 and DMF4 TCR-T cells showed similar activation profiles (Figure 4G); however, the MEL5 TCR expressing T cells were more responsive to HLA A*02:01^*+*^ melanoma lines MM909.24, MEL526, and MEL624^26^ than those transduced with the DMF4 TCR (Figure 4H). These data suggest that a multipronged TCR is potentially more sensitive to cancer cells expressing multiple antigens than a regular monospecific TCR and that recognition of multiple TAA by a single TCR may limit cancer immunoevasion.

### Melan A, BST2, and IMP2 peptides can each prime T cells with the other specificities

**Figure 5:**
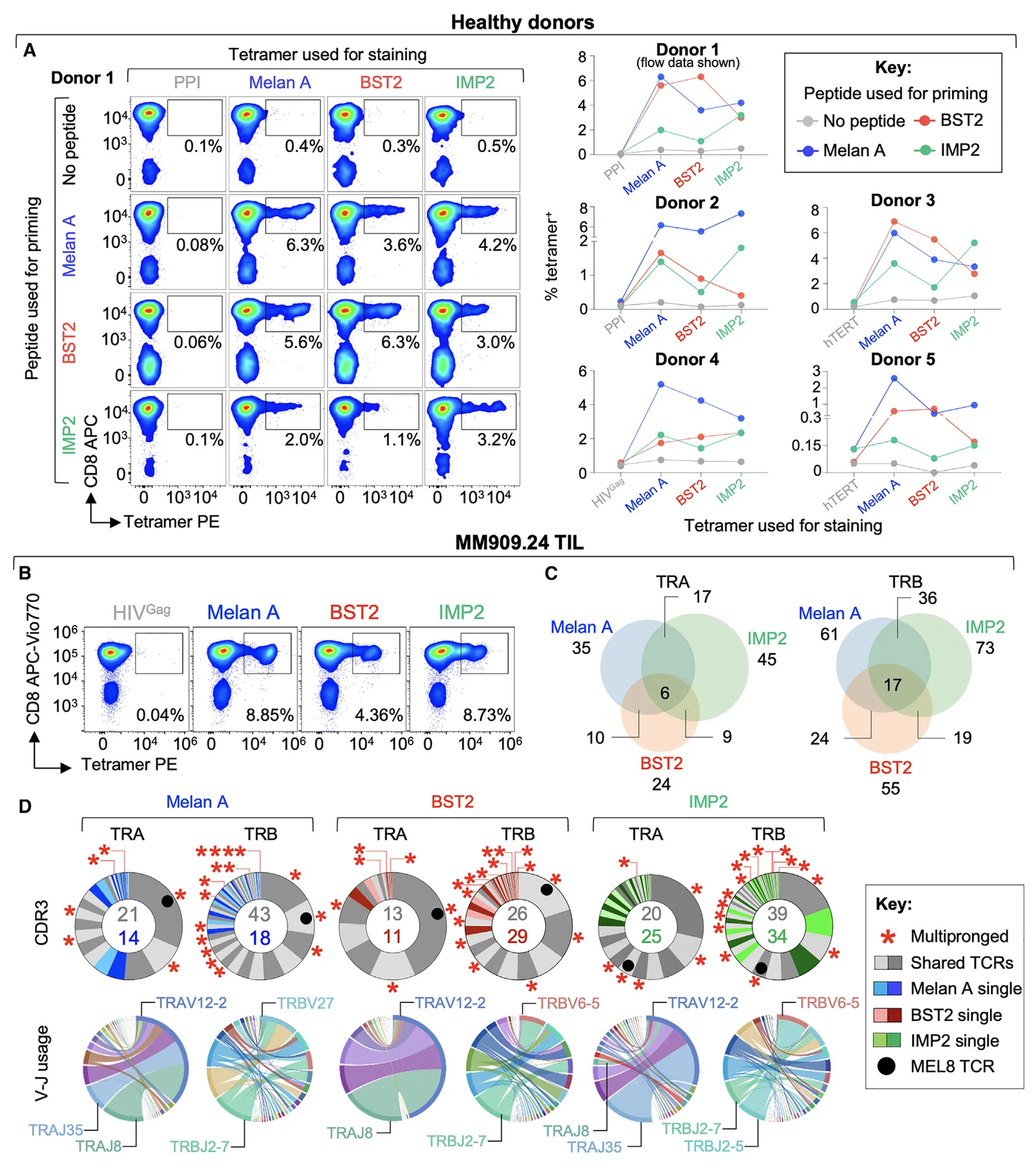
Each of the multipronged epitopes can prime T cells with the other specificities and multipronged TCRs are abundant in melanoma TILs (A) Priming of CD8 T cells (4 weeks) from healthy HLA A*02^+^ donors with Melan A_26–35_ (EAAGIGILTV), BST2_22–31_ (LLLGIGILVL), or IMP2_366–376_ (NLSALGIFST) peptides, then stained with multipronged and irrelevant tetramers (human telomerase reverse transcriptase_540–548_ (hTERT), preproinsulin_15–24_ (PPI), or HIV-Gag_77–85_). Assay schematic Figure S8A. (B) Staining of TIL (passage-1) from melanoma patient MM909.24 with multipronged and irrelevant (HIV-Gag_77–85_)^20^ tetramers. (C) Venn diagram of alpha (TRA) and beta (TRB) TCR chains from tetramer sorts in (B). (D) TCR summary of tetramer sorted cells from (B).Complementarity determining regions (CDRs) 3: gray segments indicate appearance in at least 2 of the tetramer populations. Red asterisks indicate presence in all three populations. Colored segments indicate CDR3s that are unique to indicated sort. The MEL8 TCR (black circle) appeared in all sorts. Variable (V) and joining (J) usage (arcs on right and left respectively) with dominant genes annotated. TCRs/CDR3s in Table S5.

We next examined whether each antigen could prime T cells that saw the other epitopes from the peripheral blood mononuclear cells (PBMCs) of healthy donors to test whether many, or all, individuals possessed naive T cell clonotypes that exhibited this multipronged property. Purified CD8 T cells were left unprimed, or primed with individual Melan A_10_, BST2_10_, or IMP2_10_ peptides for 4 weeks, then each culture stained with Melan A_10_, BST2_10_, and IMP2_10_ tetramers (schematic, Figure S8A). We found evidence that multipronged T cells that bind Melan A_10_, BST2_10_, and IMP2_10_ tetramers could be primed from the PBMC of all eight individuals we tested with five example experiments shown in Figure 5A. Overall, BST2_10_ or Melan A_10_ seemed to be more potent than IMP2_10_ for T cell priming. We conclude that most individuals possess T cells that have potential to simultaneously recognize epitopes from Melan A, BST2, and IMP2 proteins, although such clonotypes seem to be in the minority of those with capacity to respond to the Melan A_10_, BST2_10_, or IMP2_10_ epitopes.

### Multipronged T cells are safe *in vivo*

The potent and broad tumor-reactivity of multipronged T cells makes their TCRs very attractive candidates for use in cancer immunotherapy. While Melan A TCRs have been used in TCR-T therapies and induced cancer regression,^13,28^ there are no such data to indicate whether targeting BST2 and/or IMP2 would be safe *in vivo*. Both proteins are expressed in many cancers (Figures 2H and S5) where they are thought to play a role in tumorigenesis and are associated with poorer prognoses.^29–42^ However, other data suggest that some healthy tissues might express BST2 and/or IMP2, suggesting that therapeutic targeting of such antigens could be dangerous. The MEL8 clonotype isolated from the TIL used to successfully treat patient MM909.24 persists in the blood of the patient long after complete remission.^43^ The persistence of this clonotype during and after successful cancer treatment suggests that it could have played a role in keeping patient MM909.24 cancer free. The MEL8 clonotype was present in blood after TIL infusion without indications of pathology due to BST2 or IMP2 targeting indicating that this activated T cell clonotype must have been safe *in vivo*. To further examine this aspect, we stained the TIL infusion product used to successfully treat patient MM909.24 with Melan A_10_, BST2_10_, and IMP2_10_ tetramers, with 8.85%, 4.36%, and 8.73% of the TIL stained with the respective tetramers (Figure 5B) confirming that the patient was infused with an activated T cell product containing T cells with each specificity. We conclude that BST2_10_ and IMP2_10_-specific T cells exhibit a promising safety profile even when transfused into cancer patients in activated form in very large number (>4.7 × 10^9^ and 9.6 × 10^9^ activated T cells with each specificity were transferred to patient MM909.24 based on one-dimensional tetramer staining in Figure 5B). We next sequenced the T cells within the MM909.24 TIL product that stained with Melan A_10_, BST2_10_, or IMP2_10_ tetramers to build a picture of which TCRs in each population exhibited the ability to recognize more than one of these antigens (Figures 5C and 5D). To ensure that the cleanest antigen-specific populations where sequenced, TIL were stained with each tetramer in two dimensions as shown in Figure S8B. This double staining approach reduced the fluorescence intensity with each reagent and lowered the overall fraction of TIL sorted for sequencing to 4.91%, 0.67%, and 0.44% of total TIL for Melan A_10_, BST2_10_, and IMP2_10_ antigens, respectively. Between them, the tetramer double populations in Figure S8B expressed 75 TCRα and 126 TCRβ chains (Figure 5D, V and J gene usage, and CDR3s in Table S5). The MEL8 clonotype dominated the cell populations staining with Melan A_10_ and BST2_10_ tetramers and was present within the cells that stained with IMP2_10_. Remarkably, T cell clonotypes with 6 TCRα and 17 TCRβ within the MM909.24 TIL infusion product also stained with all three tetramers with these clonotypes making up the majority of the response within these TIL (Figures 5C and 5D). The dominance of multipronged clonotypes within this TIL population and the persistence of the MEL8 clonotype during and after complete tumor remission suggest that cells of this type may have played an important role in cancer clearance and further underscores the safety of T cells that respond to BST2_10_ and IMP2_10_ epitopes. As both BST2 and/or IMP2 are highly expressed by most cancer types (Figures 2H and S5) and T cells with these specificities appear to be safe when infused *in vivo* in the autologous TIL setting, we next wondered whether they might be present in patients with other cancer types.

### Multipronged T cells in non-melanoma cancer patients

**Figure 6:**
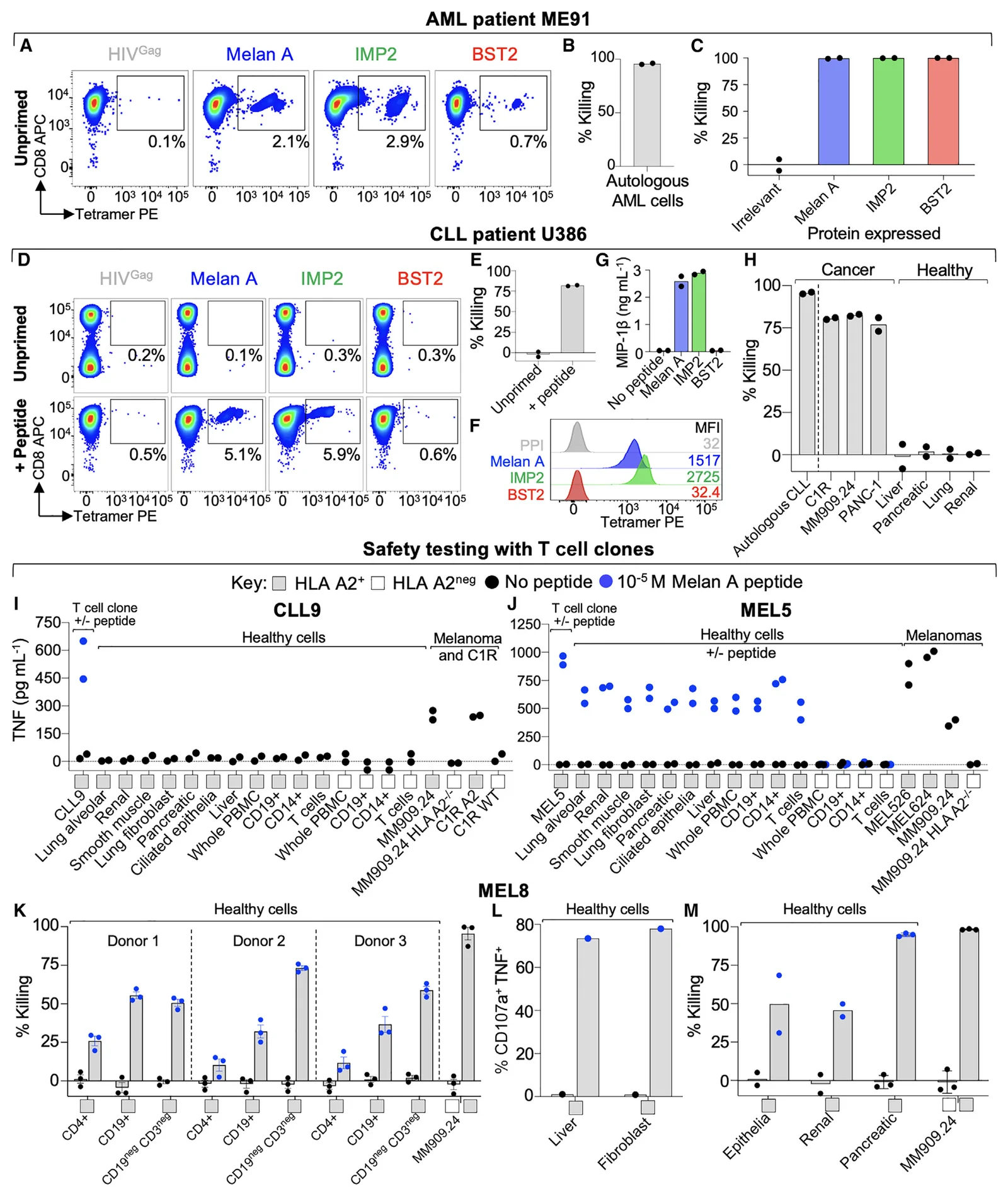
Multipronged T cells are present in leukemia patients and do not recognize healthy cells (A) CD8 T cells from AML patient ME91 were cultured *ex vivo* for 4 weeks without antigen then stained with Melan A_26–35_ (EAAGIGILTV), BST2_22–31_ (LLLGIGILVL), IMP2_367–376_ (NLSALGIFST) and irrelevant (HIV-Gag_77–85_) tetramers. (B) Overnight killing assay of the T cell line in (A) with autologous AML cancer cells at a T cell to cancer cell ratio of 10:1. (C) Killing assay for the T cell line in (A) versus MOLT3 cell line + HLA A*02:01, and genes encoding Melan A, BST2, or IMP2. Irrelevant gene encoding rCD2 used as a negative control. T cell to target cell ratio of 10:1. (D) CD8^+^ T cells from CLL patient U386 were left unprimed or stimulated with IMP2 peptide (4 weeks) then stained with multipronged and irrelevant (HIV-Gag_77–85_)^20^ tetramers. (E) Killing assay (24 h) of the T cell lines in (D) versus autologous CLL cancer cells at a 1:1 T cell to target cell ratio. (F) CD8^+^ T cell clone CLL9 was isolated from the IMP2 primed line shown in (D). Staining of CLL9 clone with multipronged and irrelevant preproinsulin_15–24_ (PPI)^21^ tetramers. MFI, mean fluorescence intensity. (G) Overnight activation (MIP-1β ELISA) of CLL9 with peptides at 10^−5^ M. (H) Killing assay (24 h) with CLL9 and autologous CLL cells (performed on different day), other cancer cell types (±HLA A*02:01) and healthy cell lines (3:1 T cell to target cell ratio). HLA A*02:01 dependent killing is displayed for melanoma-MM909.24, pancreatic-PANC-1, and C1R cells. (I) Activation assay (TNF ELISA) for CLL9 clone versus healthy cell lines, melanoma MM909.24 and C1R cells ± HLA A*02:01. (J) Overnight activation (TNF ELISA) for MEL5 clone versus healthy cell lines ± pulsed peptide, andmelanomas. Melanoma MM909.24 ± HLA A*02:01. (K) Killing assay (48 h) with MEL8 and immune cells from three HLA A*02^+^ donors ± pulsed peptide, and melanoma MM909.24 ± HLA A*02:01 (1:1 T cell target cell ratio). (L) MEL8 T107 assay (4 h) with healthy cell lines ± pulsed peptide. (M) Killing assay (48 h) with MEL8 versus healthy cell lines ± pulsed peptide, and autologous melanoma MM909.24 ± HLA A*02:01 (1:1 T cell target cell ratio).

We have observed expanded populations of IMP2_10_-specific T cells in the blood of other cancer patients. The pinnacle of this distortion was observed in an acute myeloid leukemia (AML) patient ME91 at the point of presentation at University Hospital Wales. CD8 T cells were isolated from this patient and cultured with cytokines for 4 weeks then stained with an IMP2_10_ tetramer, revealing that more than 1 in 50 of this patient’s T cells recognize the IMP2 epitope in the absence of any specific stimulation through the TCR (Figure 6A). A proportion of CD8 T cells in this donor also stained with Melan A_10_ (2.1%) and BST2_10_ (0.7%) tetramers (Figure 6A) suggesting that this patient might also have multipronged TCRs that can recognize these epitopes. The multipronged T cell line from this donor exhibited good killing of primary autologous AML cells (Figure 6B) and antigen presenting cells (APCs) expressing Melan A, BST2, or IMP2 individually as transgenes (Figure 6C). We also examined Melan A_10_, BST2_10_, and IMP2_10_ specific T cells in the context of chronic lymphocytic leukemia (CLL). Patient U386 derived T cells primed with IMP2_10_ peptide for 4 weeks had IMP2_10_ (8.7%) and Melan A_10_ (8.4%) populations, but no detectable BST2_10_ T cells (Figure 6D). The IMP2_10_ peptide primed T cell line killed autologous cancer cells, whereas no killing was seen for the unprimed line (Figure 6E). We produced monoclonal T cells from the patient U386 T cell line. TCR sequencing of U386-derived T cell clone CLL9 revealed *TRAV12-2* usage (Figure S8C), similar to MEL8 and MEL5. The CLL9 T cell clone from patient U386 grew better than other clones from this patient allowing further study. Akin to the T cell line that the CLL9 clone was generated from, it stained with Melan A_10_ and IMP2_10_ tetramers, but not for BST2_10_ (Figure 6F), which was confirmed in a peptide activation assay (Figure 6G). CLL9 also killed autologous CLL cells in addition to a variety of cancer cell lines, but did not kill healthy cells from the liver, pancreas, lung, or kidney (Figure 6H). We next extended this initial experiment to look at a wider range of healthy cell lines and other T cell clones. The bi-specific T cell clone CLL9 and the tri-specific T cell clones MEL5 and MEL8 all recognized or killed HLA A*02:01^+^ cancer lines but remained inert to a range of HLA A*02:01^+^ healthy cell lines (Figures 6I–6M), except in the presence of exogenously supplied Melan A_10_ peptide (Figures 6J–6M).

## Discussion

We set out to dissect what cancer-specific T cells respond to following successful TIL therapy for stage IV melanoma. The TIL infusion product use to induce a complete durable remission in patient MM909.24 responded to a range of different HLA A*02:01^+^ cancer cell lines from different tissues suggesting that the majority of the anticancer response in these TIL acted via HLA A*02:01 to target widely shared antigens rather than patient-specific, mutation-derived neoantigens. We have previously shown that the majority of the melanoma-reactive TIL from patient MM909.24 TIL recognize the HLA A*02:01-restricted Melan A_10_ epitope EAAGIGILTV.^16,43^ The dominant T cell clonotype in these Melan-A-reactive TIL, MEL8, persisted in patient blood following complete remission.^43^ Surprisingly, the MEL8 T cell clone also responded to a wide range of HLA A*02:01^+^ cancer cell lines that do not express Melan A suggesting that this T cell clone must additionally respond to a further widely expressed TAA. To identify this other epitope, we adapted our previous proven technique for predicting pathogen-derived peptides that act as superagonists for autoimmune HLA-I-restricted T cells^8–10,44^ to finding cancer epitopes. The CANTiGEN platform was successful in predicting the peptides recognized by five different HLA A*02:01-restricted T cell clones and identified that orphan cancer-specific T cell clones VB8 and GD.2.11 from the TIL infusion products used to successfully treat patients MM909.24 and MM909.11 recognized Melan A_9_ HLA A*02:01-AAGIGILTV and TAG HLA A*03:01-RLSNRLLLR, respectively. We conclude that the CANTiGEN pipeline can be used to efficiently predict the peptides of different length recognized by orphan cancer-specific T cell clones that have different HLA-restrictions. CANTiGEN can be applied to any conventional CD8 T cell or TCR regardless of the HLA restriction without the need for bespoke reagents.

We next applied the CANTiGEN pipeline using PS-CPL data generated with the MEL8 T cell clone. Surprisingly, MEL8 responded to three of the top four predicted agonist peptides: Melan A_10_, LLLGIGILVL from BST2 (BST2_10_), and NLSALGIFST from IMP2 (IMP2_10_) and stained with pHLA tetramers of all three peptides. The BST2_10_ and IMP2_10_ peptides are previously unreported. The previously described 9-mer BST2_9_ peptide^45^ was not recognized by TCRs of this study (Figures S7C–S7F). Most cancer cell lines express BST2 and IMP2, and almost all that have been looked at in detail express at least one of these antigens making multipronged TCRs like MEL8 attractive candidates for broad spectrum cancer immunotherapy approaches. To determine how an individual TCR could engage three different epitopes from different TAA we tried to solve the structure of the MEL8 TCR in complex with each antigen. Attempts to generate structures with the BST2_10_ and IMP2_10_ epitopes failed, but we were able to generate a structure with the Melan A_10_ epitope. The MEL8 TCR made more contacts with peptide residues Gly4 and Ile7 than any other residues, suggesting a hotspot binding motif. The MEL8 TCR binding mode is similar to that adopted by the Melan A_10_-specific MEL5 TCR, leading us to test whether this potent anti-melanoma T cell clone also recognized the BST2_10_ and IMP2_10_ epitopes. MEL5 responded to both peptides and stained with Melan A_10_, BST2_10_, and IMP2_10_ tetramers. The MEL5 TCR is much easier to make as a soluble molecule than the MEL8 TCR and crystallizes well. We solved the structure of the MEL5 TCR in complex with HLA A*02:01-BST2_10_ and -IMP2_10_ at 2.1 and 2.55 Å, respectively, and compared these with the MEL5 HLA A*02:01-Melan A_10_ complex.^22^ MEL5 TCR exhibits a conserved mode of interaction irrespective of which TAA epitope it is bound to, whereby hotspot residues P4, P5, and P7 on each epitope act as molecular mimics, with most hydrogen bonds formed between the TCR and peptide residues P4 and P7. Homology modeling of the MEL8 TCR suggested a similar hotspot-mediated mode of binding and highlighted the importance of molecular mimicry in TAA crossreactivity. Overall, we provide conclusive evidence that a single TCR can engage HLA A*02:01-restricted epitopes from three very different proteins expressed by cancer cells. Importantly, single T cells were able to kill cancer targets expressing each antigen endogenously indicating that all three epitopes are efficiently processed and presented in the context of HLA A*02:01. We further demonstrated that recognition of the Melan A_10_-, BST2_10_-, and IMP2_10_-specific epitopes was additive suggesting that endogenous expression of more than one of these proteins will lead to more potent T cell activation.

We next showed that a minority of the T cells primed from PBMC of healthy donors with any of the three epitopes could crossreact with the other epitopes. This finding suggests that a minority of T cells that have the capacity to respond to Melan A_10_, BST2_10_, or IMP2_10_ can recognize the other epitopes and opens the possibility that it might be possible to specifically induce these TCRs via targeted vaccination strategies that preferentially generate multipronged T cells. Examination of the MM909.24 TIL that stained with peptide-HLA tetramers made with Melan A_10_, BST2_10_, or IMP2_10_ peptides showed that the MEL8 clonotype dominated the populations staining with the Melan A_10_ and BST2_10_ reagents. Several lines of evidence show that T cells specific for Melan A_10_, BST2_10_, or IMP2_10_ are safe *in vivo*. The most compelling evidence of safety comes from the TIL of patient MM909.24 as tetramer staining of the infusion product demonstrated that the patient received large numbers (over 9.7 × 10^9^, 4.7 × 10^9^, and 9.6 × 10^9^ activated T cells specific for Melan A_10_, BST2_10_, or IMP2_10_ were transferred into this patient based on the number of TIL that stain with each respective tetramer) without apparent adverse effects. Indeed, the MEL8 T cell clone was the dominant Melan A_10_-specific clonotype in the TIL used to treat patient MM909.24 and was expanded in the blood of patient MM909.24 in a sample taken over 18 months after complete tumor remission. Patient MM909.24 remains tumor-free over a decade after initial TIL treatment. Importantly, this result indicates that TCR-T therapy with TCRs like MEL8 might be safe and well tolerated.

We further found >2% of T cells from AML patient ME91 stained with HLA A*02:01-IMP2_10_ tetramer at the point of presentation. This is by far the highest level of cancer-specific T cells for one epitope that we have ever seen without the need for enrichment with peptide or tetramer. These T cells efficiently lysed autologous AML cells suggesting that they had been expanded by the patient’s cancer. In a similar fashion IMP2_10_-specific T cells from CLL patient U386 could kill primary autologous CLL cells and a clone from this donor, CLL9 was found to stain with both IMP2_10_ and Melan A_10_ tetramers. CLL9 was able to kill a range of other cancer types but remained inert to all healthy cell types tested; data echoed by MEL5 and MEL8, which also remained inert to healthy cells.

Overall, we demonstrate that T cells can recognize multiple different HLA-restricted epitopes on the surface of the same cancer cell and define such T cells as being multipronged. This type of recognition is similar to, but distinct from, molecular mimicry during autoimmune disease or heterologous immunity, whereby cross reactive T cells recognize unique epitopes found on different target cells (Figure S8D). Our findings raise several interesting and important points. First, the apparent safety of having large, expanded populations of IMP2_10_- and/or BST2_10_-specific T cells *in vivo* question whether these antigens are expressed at any significant level in any healthy tissue. If these antigens were expressed, processed, and presented on HLA A*02:01 then one would have expected to have seen pathology in patients receiving such T cells or that have naturally expanded populations of cancer-reactive T cells with these specificities. Second, T cells capable of recognizing multiple TAA simultaneously have potential access to more cognate ligands on the cancer cell surface than T cells bearing TCRs that do not have this capacity as demonstrated by recognition of low concentrations of the peptide acting additively to enhance recognition. Our findings suggest that multipronged T cells might be superior to those that only target cancer cells via a single antigen. Third, it will be more difficult for cancer to escape from multipronged T cells that target multiple antigens simultaneously than those more commonly observed T cells that target just a single antigen. Antigen loss is a concern during TCR-T therapies as *in vitro* experiments have shown that melanomas can lose presentation of the Melan A_10_ epitope when co-cultured with cognate T cells.^46^ Any cancer cell that loses expression of an antigen recognized by a regular, monospecific T cell will gain a selective advantage *in vivo* that would not exist in the presence of attack from a multipronged T cell (Figure S8E). Indeed, our data showing MEL8 TCR-T recognition of melanoma cells lacking expression of Melan A_10_ and BST2_10_ provides evidence that a multipronged TCR is equipped to overcome some antigen loss immuno-evasion strategies enlisted by cancer cells. The ability of MEL8 TCR-T cells to recognize multiple cancer types is attractive from a therapeutic standpoint, as once rigorous pre-clinical testing is completed, a single multipronged TCR could be employed in clinical settings to target various cancers via TCR-T therapy.

Finally, our discovery of two new epitopes from broadly expressed TAA and a concept of how some T cells can attack the same target via different HLA-associated peptides simultaneously highlights the potential worth of detailed, unbiased examination of how successful T cells recognize their targets. Current thinking often attributes complete responses to T cell checkpoint blockade and/or TIL therapy to the recognition of mutation-derived neoantigens. We believe that such neoantigen-specific T cells exist within the cohort of TIL therapy patients we have been studying as a minority of T cell clones only respond to the autologous tumor and not to other HLA-matched melanoma cell lines. However, these patient-specific responses do not appear to dominate the response to tumor in patient blood during and after complete remission in most patients. Instead, as here, most of the T cells that respond to the autologous tumor respond to other HLA-matched melanoma lines suggesting that they respond to common shared antigens. We are currently exploring a number of other examples where individual TCRs appear to respond to multiple different antigens on the surface of the same target cell so this phenomenon may yet turn out to be common across T cell immunity and provide another evolutionary benefit of T cells being so highly cross reactive to add to those that have already been suggested.^47^

### Limitations of the study

While the dominance of T cell clonotypes in MM909.24 TIL that recognize three different HLA A*02:01-restricted TAA appears unusual, it is not possible to link the presence of these potent anticancer T cells to the clearance of end-stage cancer by this patient. The large number of these multipronged T cells in the TIL infusion product used to treat patient MM909.24, and seen in patients with other cancers, suggests that such cells are safe in the autologous TIL or natural situations. Our study was unable to determine whether such TCRs would be safe in a TCR-T setting.

## Star⋆Methods

Detailed methods are provided in the online version of this paper and include the following:

- KEY RESOURCES TABLE
- RESOURCE AVAILABILITY
  ∘ Lead contact
  ∘ Materials availability
  ∘ Data and code availability
- EXPERIMENTAL MODEL AND STUDY PARTICIPANT DETAILS
  ∘ Human subjects
  ∘ Established cell lines
  ∘ Modified cell lines
  ∘ Primary cells
  ∘ T cells
- METHOD DETAILS
  ∘ Isolation of PBMCs
  ∘ Cloning and growing T cells
  ∘ Cloning established cell lines
  ∘ Peptide priming of T cells
  ∘ Flow cytometry and cell sorting
  ∘ Tetramer assembly and staining
  ∘ Intracellular cytokine staining and T107 assay
  ∘ Intracellular staining for Melan A in cancer cell lines
  ∘ Cytotoxicity assays
  ∘ Enzyme Linked Immunosorbant Assays (ELISA)
  ∘ Peptide based assays
  ∘ TCR sequencing
  ∘ Transgene Expression
  ∘ CRISPR-Cas9 Editing
  ∘ Soluble inclusion body production
  ∘ Protein refolding
  ∘ Protein purification
  ∘ pMHC monomer biotinylation
  ∘ Sitting-drop crystallization
  ∘ 3D structure determination, analysis and homology modeling
  ∘ Data display
  ∘ CANTiGEN
- QUANTIFICATION AND STATISTICAL ANALYSIS
- ADDITIONAL RESOURCES

## Star⋆Methods

### Key Resources Table

**Table:** 

REAGENT or RESOURCE	SOURCE	IDENTIFIER
Antibodies		
HLA Class I A1 A36 antibody (clone 4i72)	Abcam	Cat# ab33642: RRID:AB_742133
Mouse anti-Human HLA A2 Low Endotoxin (clone BB7.2)	Bio-Rad	Cat# MCA2090EL; RRID: AB_1605027
Mouse anti human HLA A2 FITC (clone BB7.2)	Bio-Rad	Cat# MCA2090F; RRID: AB_324186
Mouse anti human CD3 PerCP (clone BW264/56)	Miltenyi Biotec	Cat# 130-113-131; RRID: AB_2725959
REAffinity anti-human CD8 APC (clone REA734)	Miltenyi Biotec	Cat# 130-110-679; RRID: AB_2659237
Mouse anti-human CD8 APC Vio770 (clone BW135/80)	Miltenyi Biotec	Cat# 130-113-155; RRID: AB_2725983
Mouse anti-human CD14 Pacific Blue™ antibody (clone M5E2)	BioLegend UK Ltd	Cat# 301828; RRID: AB_2275670
Mouse anti-human CD19 Pacific Blue™ antibody (clone HIB19)	BioLegend UK Ltd	Cat# 302232; RRID: AB_2073118
TNF*α* PE-Vio770, human (clone cA2)	Miltenyi Biotec	Cat# 130-120-492; RRID: AB_2784483
CD107a (LAMP-1)-PE, human (clone H4A3)	BD Biosciences	Cat# 555801: RRID: AB_396135
Mouse anti human IFN-*γ* APC antibody (clone 45-15)	Miltenyi Biotec	Cat# 130-113-490: RRID: AB_2751116
Purified mouse anti-PE antibody (clone PE001)	BioLegend UK Ltd	Cat# 408102; RRID: AB_2168924
Purified anti-Allophycocyanin (APC) Antibody (clone APC003)	BioLegend UK Ltd	Cat# 408002; RRID: AB_345358
PerCP/Cy5.5 anti-human CD317 (BST2, Tetherin)	BioLegend UK Ltd	Cat# 348414; RRID: AB_2716043
Recombinant anti-Melan A antibody (clone EP1422Y)	Abcam	Cat# ab51061; RRID: AB_880693
Goat anti-Rabbit IgG (H+L) Cross-Adsorbed Secondary Antibody, PE	Thermo Fisher Scientific	Cat# P-2771 MP; RRID: AB_2539845
Goat Anti-Ig Polyclonal Antibody, Phycoerythrin Conjugated	BD Bioscienes	Cat# 550589: RRID:AB_393768
FITC anti-rat CD2 Antibody (clone OX-34)	BioLegend UK Ltd	Cat# 201303; RRID: AB_2228899
anti-HLA Class 1 Antigen A23, A24 antibody (clone 4i94)	United States Biological	Cat# H6098-29; RRID: AB_2228757
HLA-A3 Monoclonal Antibody APC (clone GAP.A3)	Thermo Fisher Scientific	Cat# 17-5754-42; RRID: AB_2573220
PE conjugated donkey anti-rabbit IgG, minimal cross-reactivity (Poly4064)	BioLegend	Cat# 406421; RRID: AB_2563484
Rabbit IgG Isotype control	Thermo Fisher Scientific	Cat# 10500C; RRID: AB_2532981
Bacterial and virus strains		
One Shot BL21(DE3) Chemically Competent *Escherichia coli*	Thermo Fisher Scientific	Cat# C600003
Biological samples		
MM909.24 (TIL)	National Center for Cancer Immune Therapy (CCIT) at Copenhagen University Hospital, Herlev (Denmark (DK))	N/A
MM909.11 (TIL and PBMCs)	National Center for Cancer Immune Therapy (CCIT) at Copenhagen University Hospital, Herlev (Denmark)	N/A
MM909.15 (TIL)	National Center for Cancer Immune Therapy (CCIT) at Copenhagen University Hospital, Herlev (Denmark)	N/A
AML ME91 PBMCs	Cardiff Haematology Acute Myeloid Leukaemia Research Unit	N/A
CLL U368 PBMCs	Cardiff Haematology Acute Myeloid Leukaemia Research Unit	N/A
PBMC from healthy donors	Welsh Blood Service, Velindre NHS Trust	N/A
Chemicals, peptides, and recombinant proteins		
Synthetic peptide EAAGIGILTV	Peptide Protein Research Ltd	N/A
Synthetic peptide LLLGIGILVL	Peptide Protein Research Ltd	N/A
Synthetic peptide NLSALGIFST	Peptide Protein Research Ltd	N/A
Synthetic peptide ILAKFLHWL	Peptide Protein Research Ltd	N/A
Synthetic peptide SLYNTVATL	Peptide Protein Research Ltd	N/A
Synthetic peptide FILPVLGAV	Peptide Protein Research Ltd	N/A
Synthetic peptide SLSKILDTV	Peptide Protein Research Ltd	N/A
Synthetic peptide YLEYRQVPG	Peptide Protein Research Ltd	N/A
Synthetic peptide RLSNRLLLR	Peptide Protein Research Ltd	N/A
Synthetic peptide LLLGIGILV	Peptide Protein Research Ltd	N/A
Synthetic peptide AAGIGILTV	Peptide Protein Research Ltd	N/A
Synthetic peptide ALWGPDPAAA	Peptide Protein Research Ltd	N/A
Synthetic peptide QVPLRPMTYK	Peptide Protein Research Ltd	N/A
Synthetic 8mer, 9mer, 10mer, 11mer, 12mer and 13mer peptide sizing scan mixtures (see Table S2)	Pepscan Presto Ltd	N/A
Synthetic 9mer Positional Scanning Combinatorial Peptide Library (see Table S2)	Pepscan Presto Ltd	N/A
Synthetic 10mer Positional Scanning Combinatorial Peptide Library (See Table S2)	Pepscan Presto Ltd	N/A
Protein Kinase Inhibitor (Dasatinib)	Merck	Cat# SML2589
Phusion High-Fidelity DNA polymerase	Thermo Fisher Scientific	Cat# F534L
Puromycin	Invitrogen	Cat# A1113802
Amphotericin B	Merck	Cat# A2942
Ciprofloxacin	Ciproxin, Bayer, Leverkusen, Germany	Cat# 40310031-3
Protein Kinase Inhibitor Dasatinib	Axon Medchem, VA, USA	Cat# SML2589
TAPI-0	Insight Biotechnology	Cat# sc-203410
T4 DNA Ligase	Thermo Fisher Scientific	Cat# EL0011
EcoRI restriction enzyme	Thermo Fisher Scientific	Cat# FD0274
NdeI restriction enzyme	Thermo Fisher Scientific	Cat# FD0583
Thermosensitive Alkaline phosphotase	Thermo Fisher Scientific	Cat# EF0651
Tris(hydroxymethyl)aminomethane	Merck	Cat# 1083821000
Ethylenediaminetetraacetic acid	Merck	Cat# 324503-1KG
BirA kit	Avidity	Cat# BirA-RT
Isopropyl β-D-1-thiogalactopyranoside	Generon	Cat# 21530057-5
Dithiothreitol	Merck	Cat# 11583786001
Cysteamine	Merck	Cat# M9768-5G
Cystamine	Merck	Cat# C121509-25G
L-Arginine	Merck	Cat# A8094-1KG
T cell optimized HT-96 screen	Bulek et al.^48^	N/A
PACT Premier crystallization screen	Molecular Dimensions	Cat# MD1-36
Chromium-51 Radionuclide, 1mCi (37MBq), Sodium Chromate in normal saline (pH 8-10)	Perkin Elmer	NEZ030001MC
Streptavidin R-phycoerythrin conjugate (SAPE), premium grade	Thermo Fisher Scientific	Cat# 10104572
Streptavidin, Allophycocyanin conjugate, premium grade	Thermo Fisher Scientific	Cat# S32362
Remel/Oxoid Phytohaemagglutinin (PHA)	Thermo Fisher Scientific	Cat#10082333
Critical commercial assays		
RNA protect cell reagent	Qiagen	Cat# 76526
RNeasy Plus Micro kit for RNA extraction	Qiagen	Cat# 74034
SMARTer RACE 3’/5’ cDNA	Takara Clontech	Cat# 634859
Qubit dsDNA HS Assay kit	Thermo Fisher Scientific	Cat# Q32851
MiSeq Reagent Kit v2	Illumina	Cat# MS-102-2003
Anti-human CD8 magnetic microbeads	Miltenyi Biotec	Cat# 130-045-201
Human MIP1-β DuoSet ELISA	R&D Systems	Cat# DY271
Human TNF alpha DuoSet ELISA	R&D Systems	Cat# DY210
Anti-PE magnetic microbeads	Miltenyi Biotec	Cat# 130-048-801; RRID: AB_244373
LIVE/DEAD Fixable Violet Dead Cell Stain Kit (VIVID)	Thermo Fisher Scientific	Cat# L34964
Dynabeads™ Human T-Activator CD3/ CD28 for T Cell Expansion and Activation	Thermo Fisher Scientific	Cat# 10548353
CellTrace™ CFSE Cell Proliferation Kit, for flow cytometry	Thermo Fisher Scientific	Cat# C34554
FcR Blocking Reagent, human	Miltenyi Biotec	Cat# 130-059-901; RRID: AB_2892112
Deposited data		
HLA A*0201-NLSALGIFST structure	Protein Data Bank	PDB:7Q98
MEL5:HLA A*0201 -LLLGIGILVL structure	Protein Data Bank	PDB:7Q99
MEL5:HLA A*0201 -NLSALGIFST structure	Protein Data Bank	PDB:7Q9A
MEL8:HLA A*0201 -EAAGIGILTV structure	Protein Data Bank	PDB:7Q9B
HLA A*02:01 -LLLGIGILV structure	Protein Data Bank	PDB:7ZUC
Experimental models: Cell lines		
MM909.09 melanoma	National Center for Cancer Immune Therapy (CCIT-DK)	Not available
MM909.11 melanoma	CCIT-DK	Not available
MM909.12 melanoma	CCIT-DK	Not available
MM909.14 melanoma	CCIT-DK	Not available
MM909.15 melanoma	CCIT-DK	Not available
MM909.24 melanoma	CCIT-DK	Not available
MM909.37 melanoma	CCIT-DK	Not available
FM-2	CCIT-DK	RRID: CVCL_C576
FM-3	CCIT-DK	RRID: CVCL_2046
FM-6	CCIT-DK	RRID: CVCL_C581
FM-28	CCIT-DK	RRID: CVCL_C583
FM-45	CCIT-DK	RRID: CVCL_9674
FM-48	CCIT-DK	RRID: CVCL_C587
FM-55p	CCIT-DK	RRID: CVCL_C591
FM-56	CCIT-DK	RRID: CVCL_C593
FM-57	CCIT-DK	RRID: CVCL_C594
FM-72	CCIT-DK	RRID: CVCL_C602
FM-74	CCIT-DK	RRID: CVCL_C603
FM-78	CCIT-DK	RRID: CVCL_C606
FM-79	CCIT-DK	RRID: CVCL_C607
FM-81	CCIT-DK	RRID: CVCL_9678
FM-82	CCIT-DK	RRID: CVCL_C609
FM-86	CCIT-DK	RRID: CVCL_C610
FM-88	CCIT-DK	RRID: CVCL_9679
FM-92	CCIT-DK	RRID: CVCL_C615
FM-93/2	CCIT-DK	RRID: CVCL_C617
FM-95	CCIT-DK	RRID: CVCL_C618
624-mel	Locally sourced	RRID: CVCL_8054
526-mel	Locally sourced	RRID: CVCL_8051
SKMel-3	Locally sourced	RRID: CVCL_0550
SKMel-28	Locally sourced	RRID: CVCL_0526
MDA-MB-231	Locally sourced	RRID: CVCL_0062
MONO-MAC-1	DSMZ	Cat# ACC252; RRID: CVCL_1425
SiHa	Locally sourced	RRID: CVCL_0032
A2780	Locally sourced	RRID: CVCL_0134
PC-3	Locally sourced	RRID: CVCL_0035
COLO 205	Locally sourced	RRID: CVCL_0218
ACHN	Cell Line Service (CLS)	Cat# 300117; RRID: CVCL_1067
C1R	Locally sourced	RRID: CVCL_6374
MOLT3	Locally sourced	RRID: CVCL_0624
T2	Locally sourced	RRID: CVCL_2211
AsPC-1	Locally sourced	RRID: CVCL_0152
MIA PaCa-2	Locally sourced	RRID: CVCL_0428
PANC-1	Locally sourced	Cat# CRL-1469;RRID: CVCL_0480
HEK293T	Locally sourced	RRID: CVCL_0063
CD8^+^ TCR^neg^ Jurkat cells	Professor Peter Steinberger	Muller et al.^49^
CD8 T cell clone MEL8	This paper	N/A
CD8 T cell clone GD.FIL.6/30	Theaker et al.^50^	N/A
CD8 T cell clone ILA1	Laugel et al.^51^	N/A
CD8 T cell clone Lucky.6.NYBR1	This paper	N/A
CD8 T cell clone MAGE-72	This paper	N/A
CD8 T cell clone ST.64.NYBR1	This paper	N/A
CD8T cell clone GD.2.11	This paper	N/A
CD8 T cell clone MEL11	This paper	N/A
CD8 T cell clone MEL25	This paper	N/A
CD8 T cell clone MEL31	This paper	N/A
CD8 T cell clone MEL5	Cole et al.^24^	N/A
CD8 T cell clone CLL9	This paper	N/A
Human renal epithelial cells (HREpiC)	ScienCell Research Laboratories	Cat# 4120
Human pancreatic stellate cells	ScienCell Research Laboratories	Cat# 3830
Human pulmonary alveolar epithelial cells	ScienCell Research Laboratories	Cat# 3200
Human pulmonary fibroblasts	ScienCell Research Laboratories	Cat# 3300
Human hepatocytes	ScienCell Research Laboratories	Cat# 5200
Human colonic smooth muscle cells	ScienCell Research Laboratories	Cat#2940
Human non-pigmented ciliary epithelial cells	ScienCell Research Laboratories	Cat#6580
Oligonucleotides		
T7 Forward primer: TAATACGACTCACTATAGGG	Eurofins	N/A
Universal Primer A (forward): TAATAC GACTCACTATAGGGCAA GCAGTGG TATCAACGCAGAGT	Takara Clontech	Cat# 634859
TCR-Cb-R1 (reverse): GAGACCCTCAGGCGGCTGCTC	Eurofins; Rius et al.^52^	N/A
TCR-Ca-R1 (reverse): CCATAGACCTCATGTCTAGCACAG	Eurofins; Rius et al.^52^	N/A
Oligo for MART-1 For	This paper	N/A
Oligo for MART-1 Rev	This paper	N/A
Oligo for BST2 crRNA	This paper	N/A
Oligo for HLA A*02:01 crRNA	This paper	N/A
Oligo for CCL4 crRNA	This paper	N/A
Oligo for HLA A*02:01 crRNA	This paper	N/A
NEB-i5o primers (Forward)	Table S2	N/A
NEB-i7o primers (Reverse)	Table S2	N/A
Recombinant DNA		
Transfer plasmid backbone :pELNS	pELNS plasmid kindly provided by Dr. James Riley	University of Pennsylvania
Transfer plasmid backbone: pLentiCRISPR v2	Feng Zhang (Addgene plasmid # 52961; http://n2t.net/addgene:52961)	RRID: Addgene_52961
Transfer plasmid backbone: Snap Fast (pSF)	Oxgene	Custom assembly according to the STAR Methods, with order reference ORD100677.
Transfer Plasmid: Melan A-rCD2 pELNS	This paper	N/A
Transfer Plasmid: BST2-rCD2 pELNS	This paper	N/A
Transfer Plasmid: IMP2-rCD2 pELNS	This paper	N/A
Transfer Plasmid: Collagen-rCD2 pELNS	Dolton et al.^53^	N/A
Transfer Plasmid: DMF4 TCR-rCD2 pELNS	This paper	N/A
Transfer Plasmid: MEL5 TCR-rCD2 pELNS	This paper	N/A
Transfer Plasmid: MEL8 TCR-rCD2 pELNS	This paper	N/A
Transfer Plasmid: HLA A2 B2M single chain-rCD2 pSF	This paper	N/A
Envelope Plasmid: pMD2.G	Didier Trono (Addgene plasmid # 12259; http://n2t.net/addgene:12259)	RRID: Addgene_12259
Packaging Plasmid: pMDLg/pRRE	DidierTrono (Addgene plasmid # 12251; http://n2t.net/addgene:12251)	RRID: Addgene_12251
Packaging Plasmid: pRSV-Rev	DidierTrono (Addgene plasmid # 12253; http://n2t.net/addgene:12253)	RRID: Addgene_12253
Transfer Plasmid: MART-1-pLentiCRISPR v2	This paper	N/A
Packaging Plasmid: psPAX2	DidierTrono (Addgene plasmid # 12260; http://n2t.net/addgene:12260)	RRID: Addgene_12260
Expression Plasmid: pGEM-T	Promega	Cat# A3600
pGEM-T truncated-HLA A*02:01 (with and without a biotin tag)	Garboczi et al.^54^	N/A
pGEM-T Beta-2 microglobulin	Garboczi et al.^54^	N/A
Software and algorithms		
MixCR v3.0.13	MiLaboratory LLC	MiXCR, RRID:SCR_018725
VDJviz	https://vdjviz.cdr3.net/	N/A
Graphpad PRISM v9.0.0	Graphpad	GraphPad Prism, RRID:SCR_002798
FlowJo	FlowJo	FlowJo, RRID:SCR_008520
PyMol 2.3.4	Schrodinger	PyMOL, RRID:SCR_000305
CCP4 7.1	Science and Technology Facilities Council	CCP4, RRID:SCR_007255
Phaser 2.7	Phoenix Online	Phaser, RRID:SCR_014219
Win-COOT 0.9.6	Science and Technology Facilities Council	COOT, RRID:SCR_014222
REFMAC 5.8	Science and Technology Facilities Council	REFMAC5, RRID:SCR_014225
Modeller 10.2	University of California at San Francisco; California; USA	RRID:SCR_008395
MATLAB	Mathworks	RRID:SCR_001622
CANTiGEN	This paper	https://doi.org/10.5281/zenodo.8052762
Other		
FACS Aria II	BD Biosciences	643178
ACEA NovoCyte 3005 with NovoSampler pro	ACEA, Agilent	N/A
BD FACS Canto II	BD Biosciences	N/A
Vivaflow 200 30000 MWCO PES	Sartorius	Cat# VF20P2
MiSeq	Illumina	SY-410-1003
Cellulose Nitrate Membrane Filters	Sartorius	Cat# 11306-47-N
Rock Imager 2	Formulatrix	N/A
AKTA Pure 25 L	Cytiva	29018226
Superdex 200 increase 10/300 size exclusion column	Cytiva	Cat# 1518208
POROS 50 HQ Anion Exchange column	Thermo Fisher Scientific	Cat# 15662135
Amicon Ultra-15 Centrifuge Filter Unit	Merck	Cat# UFC901024
Crystal Gryphon Liquid Handling System	Art Robbins Instruments	Cat# 620-1000-10
HiTrap Butyl HP Hydrophobic interaction column	Cytiva	Cat# 28411005
MicroBeta2® Microplate Counter with 2 detectors	Perkin Elmer	Cat# 2450-0020

### Resource Availability

#### Lead contact

Further information and requests for resources and reagents should be directed to and will be fulfilled by the lead contact, Andrew K. Sewell (sewellak@cardiff.ac.uk). A list of critical reagents (key resources) is included in the key resources table.

#### Materials availability

Information and requests for resources and reagents may be directed to lead contact, Andrew K. Sewell (sewellak@cardiff.ac.uk). Any patient-related materials that can be shared will be released via material transfer agreement and data processing agreements.

### Experimental Model and Study Participant Details

#### Human subjects

Tumor infiltrating lymphocytes and autologous cancer cell lines from stage IV metastatic melanoma patients MM909.11 (male, age 41), MM909.15 (female, age 48) and MM909.24 (male, age 56), who underwent rapid TIL therapy at the National Center for Cancer Immune Therapy (CCIT) at Copenhagen University Hospital, Herlev, Denmark (DK). PBMCs from MM909.11 were also used for the study to make LCLs. The patients had been enrolled on to the clinical trials for “Adoptive Transfer of Tumor Infiltrating Lymphocytes Therapy” (NCT00937625). The Ethical Committee of the Capital region of Denmark, the Danish Data Protection Agency, and the Danish Medical Agencies (EudraCTno. 2008-008141-20) approved the study and all patients signed a written consent form.^7,55^ Appropriate material transfer agreements and data processing agreements are in place. Acute myeloid leukaemia (AML) patient ME91 (male, age 32, Caucasian) and chronic lymphocytic leukaemia (CLL) patient U386 (female, age) were sourced from through a collaboration with clinicians at the University Hospital of Wales (UHW), Cardiff. Ethical approval for samples from leukaemia patients was granted and consent taken under the National Health Service Research Ethics Committee approved projects 17/LO/1566 and South East Wales Research Ethics Committee 13/WA/0346. Blood samples from healthy donors were sourced from the Welsh Blood Service (Pontyclun, Wales, UK) and ethical approval granted by the School of Medicine Research Ethics Committee (reference 18/56). Blood and cells derived thereof were handled in accordance with Cardiff University guidelines in alignment with the United Kingdom Human Tissue Culture Act 2004.

#### Established cell lines

Cell lines were regularly tested for mycoplasma and cultured at 37°C with 5% CO_2_ in R10 (RPMI 1640 media supplemented with 10% heat-inactivated foetal bovine serum (FBS), 100 U mL^-1^ penicillin, 100 µg mL^-1^ streptomycin and 2 mM L-Glutamine), D10 or D10-F12 (as for R10 but with DMEM or DMEM-F12 respectively). Adherent cell lines were passaged when 50-80% confluent by detachment from tissue culture flasks with D-PBS and 2 mM EDTA. Suspension cell lines were passaged at least once a week by 1:5-1:50 dilution. Cutaneous melanoma lines (FM) were provided by the CCIT-DK or sourced locally and cultured in R10: FM2, FM3, FM6, FM28, FM45, FM48, FM55p, FM56, FM57, FM72, FM74, FM78, FM79, FM81, FM82, FM86, FM88, FM92, FM93/2, FM95, Mel-624, Mel-526, SK-MEL3 and SK-MEL-28. Melanoma lines from patients MM909.11, MM909.12, MM909.14, MM909.15, MM909.24 and MM909.37 were generated from metastatic lesions at the CCIT-DK^7^ and cultured as above. Cancer cell lines from breast (MCF7/R10 and MDA-MB-231/R10), colon (COLO205/R10), pancreas (AsPC-1/R10, PANC-1/D10-F12 and MIA PaCa-2/R10), ovaries (A2780/D10), prostate (PC-3/D10) and cervix (SiHa/D10) were sourced locally, cultured as adherent cells in R10, D10 or D10-F12 as indicated for each cell line. Kidney cancer cell line ACHN from Cell Line Service (Baden-Wuerttember, Germany) were cultured in D10 as adherent cells. Suspension cell lines were cultured in R10 and included the following: MOLT-3 (acute lymphoblastoid leukaemia), C1R (derived from a lymphoblastoid cell line), T2 (lymphoma derived) and B95.8 (lymphoblast from a cotton-top tamarin monkey) were sourced locally, and TCR^neg^ CD8^+^ Jurkat cells a kind gift from Professor Peter Steinberger.^49^ MONO-MAC-1 (acute myeloid leukaemia) were from DSMZ (Braunschweig, Germany) and cultured as suspension cells in R10 supplemented with 1 mM sodium pyruvate and 1X MEM non-essential amino acids. HEK293Ts were sourced locally and used as packaging cell line for lentivirus production and cultured in D10. A lymphoblastoid cell line (LCL) from patient MM909.11 was generated for the purpose of this study, using B95.8 cell line supernatant containing EBV as previously described^56^ and cultured as suspension cells in R10. Cell line authenticity was checked regularly; based on imagery of morphology and descriptions of growth from the ATCC (https://www.atcc.org) and/or DSMZ (https://www.dsmz.de); by staining with HLA -A*02 (A2) (BB7.2, BioLegend), -A*03 (A3) (GAP.A3, Thermo Fisher Scientific, Waltham, MA, USA) and/or -A*24 (A24) antibodies (4i94, US Bio, with goat anti-mouse Ig multiple adsorbed secondary antibody, BD Biosciences); by expression of CD4, HLA-DR, CD19, HLA class I; and/or a unique property of the cell line. ‘FM’ and ‘SK’ melanomas (A2^+^ or A2^neg^), Mel-526 and Mel-624 (A2^+^ and able to activate Melan A specific T cells), melanoma MM909.24 (A2^+^ A3^neg^ A24^neg^ and ~10% suspension cells), melanoma MM909.11 (A2^neg^ A3^+^ A24^neg^ slow growing), melanoma MM909.15 (A2^neg^ A3^+^ A24^neg^ fast growing), melanoma MM909.14 (A2^neg^ A3^neg^ A24^neg^), melanoma MM909.12 (A2^neg^), melanoma MM909.37 (A2^+^ A3^neg^ A24^neg^), MCF7 (A2^+^ A3^neg^ A24^neg^), MDA-MB-231 (A2^+^ A3^neg^ A24^neg^), COLO205 (A2^+^ A3^neg^ A24^neg^ and a mix of suspension and adherent cells), AsPC-1 (A2^neg^ A3^neg^ A24^neg^), PANC-1 (A2^+^ A3^neg^ A24^neg^), MIA PaCa-2 (A2^neg^, A3^neg^, A24^+^), A2780 (A2^neg^ A3^neg^ A24^neg^), PC-3 (A2^neg^ A3^neg^ A24^+^), SiHa (A2^neg^ A3^neg^ A24^+^), ACHN (A2^neg^ A3^neg^ A24^neg^), MOLT-3 (A2^neg^ A3^neg^ A24^neg^ CD4^+^ CD19^neg^), T2 (A2^low^ HLA DR^neg^ CD19^+^ and able to upregulate A2 with exogenous A2 restricted peptides), C1R (HLA class I^neg^ and able to activate EBV specific T cells when transduced with HLA A*02:01), MONO-MAC-1 (A2^neg^ A3^+^ A24^neg^ HLA DR^+^ CD19^neg^), HEK293T (able to produce lentivirus), MM909.11-LCL (A2^neg^ A3^+^ A24^neg^ CD19^+^ CD3^neg^ CD8^neg^ CD4^neg^ and undergoes repeated divisions) and B95.8 (becomes adherent when treated with PMA and produced EBV capable of immortalising B-cells).

#### Modified cell lines

Some of the cell lines were modified by expression of transgenes or by gene ablation using CRISPR-Cas9. HLA A*02:01 was expressed in HLA A*02:01^*neg*^ cells MOLT-3, PC-3, A2780, C1R, MIA PaCa-2, SiHa, AsPC-1, ACHN and MIA PaCa2. *MLANA, BST2, IGF2BP2-Isoform 1* (IMP2), *COL6A2* (Collagen type VI alpha 2 chain) or rat CD2 were individually expressed in MOLT-3 and co-expressed with HLA A*02:01. B2M was knocked-out from PANC1, MDA-MB-231 and MONO-MAC-1 and HLA A*02:01 expressed from a single chain B2M-HLA A*02:01 construct. Expression of HLA A*02:01 and *BST2* was ablated using CRISPR-Cas9 in melanoma MM909.24 and HLA A*02:01 in COLO205. *CCL4* (MIP-1β) was knocked-out from the LCL line from patient MM909.11. Modified cells were cultured in the same media as the parental cell line. Knock-out cell lines were cloned by limiting dilution and transgene transduced cells maintained as enriched lines.

#### Primary cells

Organ and tissue derived normal/healthy cells and their proprietary culture media were obtained from Sciencell Research Laboratories (Carlsbad, CA, USA) and undergo senescence if kept in culture for extended periods. Colonic smooth muscle (SMCM 1101 media, cat. #1101), non-pigmented bronchial ciliary epithelia (EpiCM media cat. #4101), hepatocyte (HM media, cat. #5201), pulmonary alveolar epithelia (AEpiCM media, cat. #3201), renal epithelia (EpiCM media cat. #4101) and pancreatic stellate cells (SteCM media, cat. #5301). MRC5s (fibroblast) were sourced locally and cultured in EMEM (Thermo Fisher Scientific), supplemented as for R10. Blood derived normal cells were generated in-house by CD19 (B-cells) or CD14 (monocytes) positive, or CD3 (T cells) negative magnetic selection, according to the manufacturer’s instructions (Miltenyi). Purity of isolated cells was checked prior to assay with a viability stain (VIVID) and panel of antibodies: CD3, CD4, CD8, CD14, CD19 and CD56 (all antibodies from Miltenyi). Primary AML cancer cells were defrosted the day before assay and cultured in R20 (as for R10 with 20% FBS) supplemented with 1X NEAA, 1 mM sodium pyruvate, 10 mM HEPES and 2 ng mL^-1^ of GM-CSF. AML blasts were phenotyped with antibodies for CD3, CD19, CD15, CD34, CD38, CD45, CD90, CD96, CD117and CD123. Primary CLL cancer cells were co-cultured with a mouse fibroblast line (NTL) expressing CD40L, which had been irradiated (750 cGy) and cultured at 2 x10^6^ mL^-1^ for 24h in D10 supplemented with 5 ng mL^-1^ of IL-4 prior to addition of 2 x10^6^ mL^-1^ of CLL PBMC^57^ CLL cells were cultured for up to 14 d then removed from the fibroblast layer, assessed for viability and phenotyped with CD3 and CD19 antibodies.

#### T cells

T cell clones, TILs and TCR transduced CD8 T cells were cultured in T cell media (as for R10, supplemented with 20IU or 200IU mL^-1^ of IL-2 (Aldesleukin, Proleukin; Prometheus), 25 ng mL^-1^ IL-15, 10 mM HEPES, 1 mM Sodium pyruvate and 1X MEM NEAA (all Life Technologies)). T cells were expanded using irradiated allogeneic PBMCs from three buffy coats sourced from the Welsh Blood Service and 1 µg mL^-1^ Phytohaemagglutinin (PHA) (Thermo Fisher Scientific). Clones were obtained by limiting dilution in 96-U well plates. HLA A*02:01-restricted T cells clones GD.FIL.6/30, ILA1, MEL5, Lucky.6.NYBR1, MAGE-72 and ST.64.NYBR1 and were grown from healthy donors. GD.FIL.6/30 (peptide FILPVLGAV, protein P-Cadherin, residues 655-663) was obtained using T cell libraries.^50^ ILA1 (ILAKFLHWL, human telomerase reverse transcriptase, residues 540-548) was isolated from peptide stimulated lines by sorting on activation markers CD25/CD69^51^ MEL5 (EAAGIGILTV, Melan A, residues 26-35) was cloned after ELAGIGILTV tetramer sorting from ELAGIGILTV primed lines.^24^ Lucky.6.NYBR1 and ST.64.NYBR1 (SLSKILDTV, NY-BR-1, residues 904-912), and MAGE-72 (YLEYRQVPG, MAGE A3, residues 240-248) were cloned from cognate peptide primed lines following magnetic sorting of peptide activated cells with TNF and IFNγ capture kits (Miltenyi Biotec). Clones MEL11, MEL25 and MEL31 were isolated from the TILs of melanoma patient MM909.24 using HLA A*02:01-EAAGIGILTV tetramer. For peptide discovery, clones MEL8, VB8 and GD.2.11 were obtained by limiting dilution of TILs from melanoma patients MM909.24 (MEL8 and VB8) and MM909.11 (GD.2.11), followed by screening for reactivity towards autologous melanoma. Clones from MM909.24 were then screened for binding of EAAGIGILTV tetramer, which stained the MEL8 clone. CLL9 was cloned from IMP2 peptide (NLSALGIFST, residues 367-376) primed lines generated from CLL patient U386. Melan A (EAAGIGILTV), BST2 (LLLGIGILVL, residues 22-31) and IMP2 (NLSALGIFST) peptide primed lines from donors 1-5 were generated from healthy donor PBMC donated to the Welsh Blood Service. The Melan A and IMP2 line from AML patient ME91 was generated by culturing magnetically purified CD8 T cells (Miltenyi Biotec) in priming media (as for T cell media with 20 IU mL^-1^ IL-2 and no IL-15) for 2 weeks, then in T cell media with 200IU mL^-1^ IL-2 and 25 ng mL^-1^ of IL-15 for a further 2 weeks. The Melan A and IMP2 peptide line for CLL patient U386 was generated in the same manner as for ME91, but with the addition of 25 µM of the IMP2 NLSALGIFST peptide when the priming was set-up. TCR transduced T cells were generated from magnetically purified CD8 T cells from PBMCs purified from blood sourced from the Welsh Blood Service.

### Method Details

#### Isolation of PBMCs

Healthy donor blood from the Welsh Blood Service was provided as EDTA-treated buffy coats, which were seronegative tested for HIV-1, HBV and HCV. The buffy coats (~50 mL) were diluted with R10 to 150 mL and rotated overnight at 11 rpm on a digital roller shaker (SRTD6, Stuart) at RT. SepMate tubes (50 mL, Stem Cell Technologies Vancouver, Canda) were used to isolate PBMCs for irradiation (3100 cGy from a caesium source) to act as ‘feeder’ cells to generate T cell or cancer cell clones, and to routinely expand TILs and T cell clones. Each diluted buffy coat was made up to 210 mL with RPMI-1640 then placed in six SepMate tubes containing 13 mL of Histopaque 1077 (Merck) and centrifuged at 1200 x g for 10 min (followed by another 2 min spin if red blood cells were present in the top chamber). The upper layer from each SepMate tube was harvested, made up to 50 mL with RPMI-1640, centrifuged at 800 x g for 10 min, then resuspended in 25 mL of standard ammonium chloride red blood cell lysis buffer and incubated for 10 min in a 37°C water bath. After lysis, cells were washed in RMPI-1640 and centrifuged at 300 x g for 5 mins to remove platelets. RBC lysis was repeated if necessary. The resulting PBMC pellet was resuspended in R10 and cells counted by trypan blue exclusion. Buffy coats and PBMCs (CLL and AML patients) from clinic that were used for priming with peptide were processed by conventional density centrifugation methods, by layering whole blood over Histopaque 1077 at a 1:1 ratio.

#### Cloning and growing T cells

For cloning, T cells were plated in a 96U well plates containing 100 µL of T cell expansion media (as above with 20 IU mL^-1^ IL-2). Cells were plated at a density of 0.5 well^-1^ with 50,000 irradiated allogeneic PBMC from 3 buffy coats and 1 µg mL^-1^ PHA. Cells were cultured for 7 days before the addition of 100 µL T cell media. On day 14, wells containing visibly growing T cell clones were either expanded or screened for tetramer staining or functionality towards cancer cells or peptide. Expansion of T cell clones or TILs was performed in either T25 flasks or wells. For T25 flasks, up to 1 x10^6^ T cells and 15 x10^6^ irradiated allogenic PBMCs from 3 buffy coats and 1 µg mL^-1^ PHA in 15 mL of T cell media per flask, which was tilted at a 45° to allow the cells to collect. On day 5 post-expansion, half the cell media was replaced with T cell expansion media. On day 7 post-expansion, cells were washed, counted, and plated in T cell culture media (as for T cell expansion media but containing 200 IU L^-1^ IL-2 and 25 ng mL^-1^ IL-15) at a density of 1.5-2 x10^6^ mL^-1^ in 48 or 24 well plates. For expansions in 24 well plates, 0.1-0.2 x10^6^ T cells were used per well, with 4 x10^6^ irradiated allogenic PBMCs from 3 buffy coats and 1 µg mL^-1^ of PHA in 2 mL of expansion media. On day 5, half the T cell expansion media was changed, and on day 7 the T cells harvested and counted and plated in T cell culture media as above. T cells were used in functional assays after day 14 post-expansion.

#### Cloning established cell lines

For the cloning of cancer and LCLs, 0.5 cells were plated well^-1^ of 96 well plates, using U-well for suspension and flat-bottom for adherent cells lines. Each well had 5,000 irradiated allogeneic PBMCs from 3 buffy coats in 100 µL of respective media. After 7 days, 100 µL of respective media was added and half of the media changed weekly thereon, until clones appeared, which were transferred to successively larger wells or flasks before testing.

#### Peptide priming of T cells

For peptide priming from healthy donors, CD8s from freshly isolated PBMCs was magnetically purified by positive selection according to the manufacturer’s instructions (Miltenyi), then primed with peptide (>90% purity, Peptide Protein Research Ltd.) as previously described.^16^ The CD8 fraction was cultured in T cell priming media (as for T cell expansion media but with no IL-15) at a density of 3-4 x10^6^ well^-1^ of a 24 well plate. The autologous CD8^neg^ fraction was pulsed for 1 h at 37°C with 25 µM peptide in R5 (as for R10 with 5% FBS) under slow continuous rotation using a MACSmix tube rotator (Miltenyi). Following irradiation (3100 cGy), peptide pulsed autologous CD8^neg^ cells were added to the CD8 cells at a density of 6-8 x10^6^ cells well^-1^, with 0.5 µg mL^-1^ of anti-CD28 antibody (Beckman Coulter Ltd., UK). The cultures were fed thrice weekly by changing half the media. On days 14-28 the cells were cultured in T cell expansion media (IL-2 and IL-15) then used for downstream analysis. For AML patient ME91, CD8 and CD19 cells were isolated by positive enrichment with a mix of magnetic beads for the respective markers (Miltenyi Biotec), then cultured in 96U well plates with 0.1 x10^6^ cells well^-1^ in 200 µL of T cell priming media and 0.5 µg mL^-1^ of anti-CD28 antibody. The cells were fed thrice weekly by changing half the media and from day 14 onwards T cell culture media was used (IL-2 and IL-15). For CLL patient U386 a mix of PE conjugated CD14 (clone REA599), CD11b (clone REA713) and CD11c (cloneMJ4-27G12) antibodies (Miltenyi Biotec) followed by separation with CD8 and anti-PE magnetic beads (Miltenyi Biotec) and cultured as for ME91, but with 25 µM of the IMP2 NLSALGIFST peptide, which was added directly to culture without pulsing the cells first.

#### Flow cytometry and cell sorting

Staining was performed in 5 mL polystyrene test tubes (1 wash per step) or 96U well plates (3 washes per step), using 0.5-1 x10^5^ cells per sample. Dead cells were detected with 2 µL of a 1:40 PBS dilution of LIVE/DEAD® Fixable Violet Dead Stain Kit (VIVID). Samples were acquired on a BD FACSCanto II (BD Biosciences, Franklin Lakes, NJ, USA) or ACEA NovoCyte 3005 with NovoSampler pro (ACEA Biosciences, Agilent, CA, US), then analyzed with FlowJo software (Tree Star Inc., Ashland, Oregon, US). Cells were gated on FSC-A versus SSC-A, single cells (FSC-A versus FSC-H), then viable cells (marker of choice versus VIVID). Cell sorting was performed on a BD FACS Aria III (BD Biosciences) by Central Biotechnology Services at Cardiff University. T cells for TCR sequencing were captured in RLT lysis buffer supplemented with 40 mM DTT or RNAprotect reagent (both from Qiagen). T cells or established cell lines sorted for culture were collected in their respective culture media supplemented with 25 µg mL^-1^ of Amphotericin B (Thermo Fisher Scientific) and 10 µg mL^-1^ of Ciprofloxacin (Ciproxin, Bayer, Leverkusen, Germany).

#### Tetramer assembly and staining

Monomeric pMHC were generated in house as described below and used to assemble tetramers as previously described.^52^ In brief, tetramers were assembled by 5 consecutive 20 min incubations of PE or APC conjugated streptavidin (both from Life Technologies) with pMHC monomer at 1:4 molar ratio, on ice in the dark. Once assembled, 1:100 protease inhibitor (set 1, Merk, London, UK) and PBS was added to a working concentration of 0.1 mg mL^-1^ (with regards to the pMHC component). Tetramers were stored at 4°C in the dark and used within 3 days of assembly. To remove aggregates, tetramers were centrifuged at full speed in a microfuge for 1 min immediately before staining samples. Tetramer staining was performed as previously described.^52,53^ Briefly, T cell clones, lines, TILs or Jurkat cells were pre-treated with 50 nM of the protein kinase inhibitor Dasatinib at 37°C for 10-30 min in 100 µL of FACS buffer (PBS with 2% FBS), followed by the addition of 0.5 µg tetramer (with respect to pMHC component) without washing or pre-chilling, and incubated for 30 min on ice and in the dark. Cells were then washed in PBS and stained with VIVID for 5 mins at room temperature. Following incubation and without washing, fluorochrome-conjugated antibodies for CD3 and CD8 were added to T cells and incubated for 20 min on ice in the dark. If Jurkats cells were being stained then an anti-rCD2 antibody was used, as rCD2 is a co-marker for TCR expression. Unconjugated anti-PE (clone PE001) or anti-APC (clone APC003) (both from BioLegend) antibodies were added with the other antibodies to stabilize staining with tetramer.^58^ Cells were then washed in PBS and used for flow cytometry. Alternatively, the cells were washed in PBS and fixed in 2% PFA for 20 mins, followed by washing with PBS. The specific details of the peptides used as peptide-HLA tetramers can be found in Table S1.

#### Intracellular cytokine staining and T107 assay

Intracellular staining for IFNγ (antibody clone 45-15, APC conjugated, Miltenyi Biotec)^59^ was performed using 3 x10^4^ TILs with cancer cells at a 1:2 ratio in 96U well plates. HLA A*02 (BB7.2, Bio-Rad) and HLA A*01 (4i72, Abcam, Cambridge, UK) blocking antibodies were added to the cancer cells at 10 µg mL^-1^ for 30 min at RT prior to adding the TILs. The cells were then stained with VIVID, anti-CD3 PerCP and anti-CD8 APC Vio770 antibodies. T107 assays (detection of TNF and CD107a) were performed as previously described.^43^ Briefly, T cells (3-10 x10^4^) were co-incubated in 96U well plates with target cells at a 1:2 ratio, then incubated at 37°C and 5% CO_2_ in R5 media containing 30 µM TNFα processing inhibitor (TAPI)-0 (Insight Biotechnology), anti-TNF PE Vio770 (clone cA2, Miltenyi Biotec) and anti-CD107a PE (clone H4A3, BD Biosciences) antibodies. Following 4–5 h incubation, cells were stained as described above with VIVID, anti-CD3 PerCP and anti-CD8 APC Vio770 or APC antibodies (all from Miltenyi Biotec).

#### Intracellular staining for Melan A in cancer cell lines

Cancer cell lines were used at 5 x10^4^ per condition in 96U well plates and stained with VIVID as described above, washed thrice with PBS, followed by permeabilization using Beckman Dickinson’s fixation and permeabilization kit according to the manufacturer’s instructions, then three washes with Perm/Wash. Rabbit anti-Melan A (EP1422Y, Abcam) and rabbit IgG isotype (Thermo Fisher Scientific) antibodies used at 1 µg mL^-1^ for 30 min on ice, followed by three washes with Perm/Wash. PE conjugated donkey anti-rabbit IgG (minimal crossreactivity, Poly4064, BioLegend) used at 0.1 µg stain^-1^ for 20 min on ice, then 3 washes with PBS.

#### Cytotoxicity assays

Chromium-51 (^51^Cr) release cytotoxicity assay was performed as previously described^58^ Briefly, 30 µCi of ^51^Cr (sodium chromate in normal saline; PerkinElmer, Waltham, MA) was use label 1 x10^6^ target cells for 1 h at 37°C, which were then washed and incubated in R10 media for a further h at 37°C to allow excess ^51^Cr to leach from the cells. After labelling, 2 x10^3^ target cells were co-incubated in 150 µL of R10 at 37°C and 5% CO2 with T cells at a T cell to target cell ratio indicated in the results. After incubation, 15 µL of the supernatants were harvested and mixed with 150 µL of Optiphase Supermix scintillatant (PerkinElmer) and measured using a MicroBeta Microplate counter (PerkinElmer). Target cells incubated alone or with 1% Triton X-100 (Merck) were used as control for spontaneous and total ^51^Cr release, respectively. All assayed samples were performed in triplicate unless stated otherwise and the following equation used to calculate the percentage killing.

$$\%\;killing\;=\;100\;-\left(\left(\frac{Target\;cell\;release\;with\;T\;cells\;-\;Spontanous\;release\;}{Target\;cell\;total\;release\;-\;Spontaneous\;release}\right)\times \;100\right)$$

Flow-based killing assays were performed as previously described.^60^ Target cells were co-incubated with and without T cells at an E:T ratios and duration indicated in the results, in 96U-well plates containing media of 50:50 T cell media with 20IU mL^-1^ IL-2 and IL-15 and the targets cells preferred media. On the day of harvest, 2 x10^4^ C1R cells were labelled with 0.1 µM CFSE (Thermo Fisher Scientific) and added to each assay well, followed by incubation with FcR blocking reagent (Miltenyi Biotec) and staining with VIVID, CD3 and CD8 antibodies. All assayed samples were performed in duplicate or triplicate. T cells and dead/dying cells were excluded from analysis, leaving viable target cells and CFSE^+^ C1Rs to calculate the percentage of killing using the following equation.

$$\%\;killing\;=\;100\;-\left(\left(\frac{with\;T\;cells:\;\%\;target\;cells÷\;\%C1R\;CFSE}{without\;T\;cells:\;\%\;target\;cells÷\;\%C1R\;CFSE}\right)\times \;100\right)$$

Alternatively, the same cancer cell line +/- HLA A*02:01 were co-incubated +/- T cells. Conditions and staining were the same as for the flow-based killing assay above but with the addition of an HLA A*02 Ab and using the equation below to calculate percentage killing:

$$\%\;killing\;=\;100\;-\;\left(\left(\frac{with\;T\;cells\;:\;HLA\;A2\;positive\;target\;cells÷\text{\%}HLA\;negative\;target\;cells}{without\;T\;cells\;\text{:}\;\text{\%}HLA\;A2\;positive\;target\;cells÷\text{\%}HLA\;negative\;target\;cells}\right)\times \;100\right)$$

#### Enzyme Linked Immunosorbant Assays (ELISA)

T cells were ‘rested’ overnight in R5 medium (R10 but with 5% FBS) to help reduce spontaneous release of chemokines and cytokines. 3 x10^4^ T cells were used in 96U well plates at 1:2 ratio with 6 x10^4^ antigen presenting cells or target cells in R5 medium. Supernatants were harvested and half-area ELISA plates used in conjunction with MIP-1β or TNF detection kits according to the manufacturer’s instructions (R&D Systems, Abingdon, UK). T cell activation towards peptide was determined by MIP-1β release, and for cancer or healthy cell targets it was determined by TNF release. All assay conditions were performed in replicate.

#### Peptide based assays

Peptide length preference of a T cell clone was determined using ‘sizing scans’.^19^ Each sizing scan mixture consists of random peptides of different lengths (X^8^, X^9^, X^10^, X^11^, X^12^ and X^13^, where X= any of the 19 proteogenic L-amino acids, excluding cysteine) (Table S2). Each mixture was pulsed on to 6 x10^4^ antigen presenting cells at ~1 mM (the molatiry of peptide library mixtures are approximate) for 2 h at 37°C and 5% CO_2_ then incubated overnight with 3 x10^4^ Tcells. Supernatants were used for MIP-1 β ELISA as described above. Positional Scanning Combinatorial Peptide Libraries (PS-CPL) were used to establish the amino acid preference at each position of a peptide.^9,10,19,61^ Nonomer and decamer PS-CPLs consist of 180 and 200 sublibraries respectively, with each sublibrary following the format OXXXXXXXX(X); whereby ‘O’ is fixed for each of the 20 proteogenic amino acids in turn, and its position moved along the peptide backbone to create the library; ‘X’ is the degenerate position with 1 of 19 proteogenic L-amino acids, excluding cysteine^19^ (Table S2). Each sublibrary was pulsed on to 6 x10^4^ antigen presenting cells at ~100 µM for 2 h at 37°C and 5% CO_2_, prior to the addition of 3 x10^4^ T cells and followed by overnight incubation at 37°C and 5% CO_2_. Supernatant was collected and assayed by MIP-1β ELISA. Individual peptides were used at a single concentration or titrated, using 3 x10^4^ T cells and 6 x10^4^ antigen presenting cells and overnight incubation and MIP-1β. In titration experiments the functional sensitivity of a T cell towards a peptide is expressed by the EC_50_. The details of specific peptides used in this study can be found in Table S1.

#### TCR sequencing

A minimum of 50,000 T cells were harvested from culture, washed in PBS and resuspended in 300 µL of RNAprotect cell reagent (Qiagen, Hilden, Germany). Flow sorted populations of T cells were captured directly in to RNAprotect cell reagent. TCR sequencing was performed as previously described.^16^ RNA extraction was carried out using the RNEasy Micro kit (Qiagen). cDNA was synthesized using the 5′/3′ SMARTer kit according to the manufacturer’s instructions (Takara Bio, Paris, France). The SMARTer approach used a Murine Moloney Leukaemia Virus (MMLV) reverse transcriptase, a 3′ oligo-dT primer and a 5′ oligonucleotide to generate cDNA templates flanked by a known, universal anchor sequence at the 5′. A Step-Out PCR was performed using a pair of primers consisting of 3’ TRAC or TRBC-specific reverse primer (Eurofins Genomics) and a 5’ universal anchor-specific forward primer (Takara Bio, Paris, France). All samples were used for the following PCR reaction: 2.5 µL template cDNA, 0. 5 µL High Fidelity Phusion Taq polymerase, 10 µL 5X Phusion buffer, 0.5 µL DMSO, 1 µL dNTP Mix (stock concentration of 10 mM of each) (all from Thermo Fisher Scientific), 1 µL of TRAC or TRBC-specific primer (10 µM stock) (Table S2), 5 µL of 10X anchor-specific universal primer (Takara Bio, Paris, France), and nuclease-free water for a final reaction volume of 50 µL (cycling conditions: 5 min at 94°C, 30 cycles of 30 s at 94°C, 30 s at 63°C for alpha chains or 30 s at 66°C for beta chains, 120 s at 72°C). Subsequently, 5 µL of the Step-out PCR products were taken for a nested PCR, using 1 µL of barcoded forward (universal) and reverse (TRAC or TRBC) primers (10 µM stock) (Eurofins Genomics) (Table S2), 0.5 µL High Fidelity Phusion Taq polymerase, 10 µL 5X Phusion buffer, 0.5 µL DMSO, 1 µL dNTP Mix (stock concetration of 10 mM each) and nuclease-free water for a final reaction volume of 50 µL (cycling conditions: 5 min at 94°C, 30 cycles of 30 s at 94°C, 30 s at 62°C, 120 s at 72°C, and a final 10 min at 72°C). The final PCR products were loaded on a 1% agarose gel and purified with the Monarch® gel extraction kit (New England Biolabs). Purified products were sequenced on an Illumina MiSeq instrument using the MiSeq v2 reagent kit (Illumina, Cambridge, UK) according to the manufacturer’s instructions. Sequence analysis was performed using MiXCR software (v3.0.7).^62^ Public TCR clonotypes were identified using the VDJdb database.^63^

#### Transgene Expression

Codon optimized genes for *MLANA* (UniProtKB Q16655), *BST2* (UniProtKB Q10589), *IMP2* (UniProtKB Q9Y6M1-2), *COL6A2* (UniProtKB P12110) and rat CD2 were synthesized by Genewiz (South Plainfield, NJ, USA) and individually cloned into the 3^rd^ generation lentiviral transfer vector backbone pELNS (kindly provided by Dr. James Riley, University of Pennsylvania, PA, USA) containing a rat CD2 (rCD2) marker gene preceded by a P2A self-cleaving sequence: XbaI-Kozak-Gene-XhoI-P2A-rCD2-SalI-Stop. HLA A*02:01 (UniProtKB P01892) was also expressed from pELNs but without the rCD2 co-marker: XbaI-Kozak-HLA A*02:01-SalI-Stop. A *B2M* (UniProtKB P61769) HLA A*02:01 single chain construct was generated using codon optimized genes and expressed from the Snap Fast plasmid (pSF) (Oxgene, Oxford Genetics Ltd, Littlemore, Oxford, UK): Xba1-Koazk-*B2M* with leader sequence-(GGGGS)^3^-HLA A*02:01 with no leader sequence-XhoI-P2A-*rCD2*-SalI-Stop. The pSF plasmid was modified by removal of a XhoI site present in the plasmid backbone and insertion of stop codons after the SalI site. TRA and TRB chains were separated by a self-cleaving T2A and expressed from pELNs: XbaI-Kozak-*TRA*-T2A-*TRB*-XhoI-P2A-rCD2-SalI-Stop. For transfection, pSF or pELNS (1.52 µg), envelope plasmid (pMD2.G; 0.72 µg) and packaging plasmids (pMDLg/pRRE; 1.83 µg and pRSV-REV; 1.83µg) were mixed in 300 µL of Opti-MEM, reduced serum medium (Thermo Fisher Scientific) followed by mixing with 1 µg µL^-1^ Polyethylenimine (PEI; Merck Group) at a 3:1 PEI: plasmid ratio. Plasmid/PEI mixtures were incubated at room temperature for 15 min, added dropwise to HEK293T cells (80% confluence in one well of a 6-well plate) and incubated at 37°C in a 5% CO_2_ humidified atmosphere. The supernatants containing lentivirus were harvested, 0.4 µm filtered, and used immediately for transduction or stored at -80°C and only defrosted once before transduction. Immortal and cancer cells were transduced by spinfection at 400 x *g* for 2 h with 5 µg mL^-1^ of polybrene, then incubated at 37°C overnight and media was replaced the following morning. Not all cell lines tolerated polybrene, so each transduction was performed +/- polybrene. HLA A*02:01 transduction was confirmed by staining with BB7.2 Ab. *BST2, IMP2, COLA6* and B2M-HLA A*02:01 transduced cells were stained with anti-rCD2 antibody (OX-34, BioLegend), with the B2M-HLA A*02:01 cells also stained with BB7.2 and for B2M (clone 2M2, BioLegend) Abs. For each TCRs transduction, 6 wells of supernatant were combined after ultracentrifugation (140,000 x g for 2 h at 4°C). T cell transduction was performed as previously described.^64^ In brief, CD8 T cells were purified from healthy donors using anti-CD8 microbeads (Miltenyi) following manufacturer’s guidelines, followed by overnight incubation with CD3/CD28 Dynabeads (Life Technologies) at 3:1 bead:T cell ratio in T cell media with 20 IU mL^-1^ of IL-2 and 25 ng mL^-1^ of IL-15. Following activation, viral transduction was performed by spin-infection at 400 x *g* for 2h with 8 µg mL^-1^ of polybrene (Sigma Aldrich) and incubated at 37°C 5% CO_2_. 12 h post-infection half the media was replaced with T cell media containing 200 IU mL^-1^ IL-2, 25 ng mL^-1^ IL-15 and 20% FBS, then 50% of media changed thrice weekly. On day 7 post-infection, transgene expression was confirmed by rCD2 expression and enrichment achieved using PE conjugated rCD2 Ab and anti-PE microbeads (Miltenyi Biotec) following manufacturer’s instructions. On day 14, rCD2 enriched T cells were expanded and maintained as described above for T cell clones and lines.

#### CRISPR-Cas9 Editing

Oligonucleotides for the single guide (sg)RNA sequences of HLA A*02:01, MLANA, BST2, and *CCL4* were designed and cloned into pLentiCRISPR v2 with puromycin resistance gene, a gift from Feng Zhang (Addgene plasmid # 52961).^65^ Lentiviral particles were generated as described above, using PEI and 3.3 µg of gRNA-containing pLentiCRISPR v2 transfer vector, 0.825 µg of pMD2.G envelope plasmid and 1.65 µg of psPAX2 packaging plasmid. Culture media was changed after 24 h. Supernatants were harvested at 48 h and 36 h post-transfection, centrifuged (400 x *g* for 5 mins) and 0.45 µm filtered. Viral transduction was performed by spin-infection at 400 x *g* for 2 hours with 8 µg mL^-1^ polybrene (Sigma Aldrich) and incubated at 37°C and 5% CO2, followed by a media change 12 h post-infection. Cells transduced with the puromycin resistance marker gene were placed under 1 µg mL^-1^ puromycin (Thermo Fisher Scientific) selection 7 d post transduction.

#### Soluble inclusion body production

Codon optimized synthetic genes for HLA A*02:01, HLA A*03:01, Beta-2-microglobulin *(B2M)*, MEL8 α TCR chain, MEL8 β TCR chain, MEL5 TCR α chain and MEL5 TCR β chain were designed and purchased from GeneArt (Thermo Fisher Scientific). Designs took the form of NdeI-Gene-Stop-Stop-EcoRI and were cloned by double digestion at 37°C with NdeI and EcoRI restriction enzymes (Thermo Fisher Scientific) for 30 min. The pGEMT vector was simultaneously digested under the same conditions with NdeI, EcoRI, and TAP (all Thermo Fisher Scientific), the reaction was stopped by heating at 65°C for 5 min. Genes were ligated into the pGEM-T vector using T4 DNA ligase and following the manufactures guidelines. TCR genes were designed to contain a non-native disulphide bond, by substituting residues 48 and 57 of the TCR α chain and TCR β chain constant domains respectively with cysteine residues, in order to produce stable soluble TCRs.^66^ The expressed protein sequence is shown in Figure S2. A biotin tag (GLNDIFEAQKIEWHE) was added to the C-terminus of the HLA A*02:01 and HLA A*03:01 constructs for the production of biotinylated pMHC monomers. All genes in the pGEM-T vector (Promega, WI, USA) were transformed into competent BL21 DE3 E. coli cells (Thermo Fisher Scientific) and plated onto Luria Broth (LB) agar plates (10 g L^-1^ tryptone (Merck), 5 g L^-1^ yeast extract (Merck), 10 g L^-1^ sodium chloride (Thermo Fisher Scientific), 15 g L^-1^ bacteriological agar (Oxoid)) containing 50 µg mL^-1^ of carbenicillin (Thermo Fisher Scientific). Successful colonies were used to inoculate 20 mL of TYP media (16 g L^-1^ tryptone, 16 g L^-1^ yeast extract, 5 g L^-1^ sodium chloride, 3.3 g L^-1^ potassium phosphate dibasic (Merck)), which was incubated at 37°C, 220 RPM overnight. These cultures were then used to inoculate 1 L of TYP media, which was incubated at 37°C, 220 RPM until the OD_450_ measured 0.5 AU cm^−1^. Protein expression was induced with 0.5 mM Isopropyl β-D-1-thiogalactopyranoside (IPTG, Generon) under the same incubation conditions for 3 h. The cultures were centrifuged for 20 min at 2786 x *g* (Eppendorf 5810R with rotor S-4-104 and 750 mL buckets) before re-suspending the pellet in 40 mL of lysis buffer (10 mM tris(hydroxymethyl)aminomethane (TRIS) pH 8.1 (Merck), 10 mM magnesium chloride (Merck), 150 mM sodium chloride, 10% v v^-1^ glycerol (Thermo Fisher Scientific)). The resuspended pellet was frozen solid at either -20°C overnight or rapidly at -80°C and then thawed at room temperature. The thawed pellet was placed on ice and sonicated at 50% power for 20 min with a 2 x 10% pulse using a Bandelin SONOPULS Ultrasonic H sonicator with a Z659169 sonicator tip (Bandelin, Berlin, Germany), 4 mg of DNase (Merck) was then added before incubation at 37°C for 2 h. The suspension was diluted with 200 mL Triton wash buffer, 50 mM TRIS pH 8.1, 100 mM sodium chloride, 2 mM Ethylenediaminetetraacetic acid (EDTA, Merck), 0.5% v v^-1^ Triton X (Merck)) then centrifuged for 20 min at 17,700 x *g* (Beckman Avanti JE with JA 10 rotor and 500 mL polypropylene bottle w/cap assembly). The resulting pellet was resuspended in another 200 mL of triton wash and the centrifugation step repeated. The pellet was then resuspended in 200 mL of resuspension buffer (50 mM TRIS pH 8.1, 100 mM sodium chloride, 2 mM EDTA) and centrifuged for 20 min at 17,700 x *g*. The resulting pellet was resuspended in 20 mL of guanidine buffer (50 mM TRIS pH 8.1, 100 mM sodium chloride 2 mM EDTA, 6 M guanidine hydrochloride (Thermo Fisher Scientific)), resulting in soluble inclusion bodies.

#### Protein refolding

Soluble inclusion bodies were chemically refolded together by dilution of denaturing conditions to produce soluble TCR and peptide:MHC (pMHC) molecules.^67^ Soluble MEL5 TCR molecules were refolded by incubating 30 mg of MEL5 TCR α chain inclusion bodies with 10 mM dithiothreitol (DTT, Merck) at 37°C for 30 min. 30 mg of MEL5 TCR β chain inclusion bodies were also incubated with 10 mM DTT at 37°C for 30 min, with incubation beginning 15 min after the MEL5 TCRα chain incubation. 0.74 g of cysteamine (Merck) and 0.83 g of cystamine (Merck) were dissolved in 1 L of TCR refold buffer (50 mM TRIS pH 8.1, 2 mM EDTA, 2.5 M urea (Merck)) at 4°C immediately prior to adding the TCR α-chain DTT mixture. 5 min later the TCR β-chain DDT mixture was added, and the solution was continuously stirred at 4°C for 6 h. Soluble pMHC molecules were refolded by incubating 30 mg of HLA A*02:01 or HLA A*03:01 inclusion bodies, 30 mg of B2M inclusion bodies, 4 mg of peptide and 10 mM DTT together at 37°C for 30 min. 0.74 g of cysteamine and 0.83 g of cystamine were dissolved in 1 L of pMHC refolding buffer (50 mM TRIS pH 8.1, 2 mM EDTA, 400 mM L-Arginine (Merck)) at 4°C immediately prior to adding the inclusion body peptide mixture and continuously stirring at 4°C for 6 h. After the 6 h incubation the MEL5 TCR or pMHC refold mixtures were dialyzed through a cellulose dialysis membrane, (Merck) in 10 mM TRIS pH 8.1 buffer (40 L per 1 L refold buffer) until the conductivity measured below 1 mS/cm. Mixtures were then filtered through a 0.45 µm cellulose nitrate filter (Sartorius AG, Göttingen, Germany) in preparation for purification. A modified version of the refold protocol above was used for MEL8 TCR. MEL8 α and MEL8 β inclusion bodies were incubated in the same way as MEL5 inclusion bodies and added to 1 L of modified TCR refold buffer (50 mM TRIS pH 8.1, 2 mM EDTA, 2.5 M urea, 1 M L-Arginine, 1 M sodium chloride) prepare with 0.74 g of cysteamine and 0.83 g of cystamine as described above and incubated at 4°C overnight. The refold was then concentrated to 250 mL using a Viva Flow 200 10 kDa MW cassette (Sartorius AG), before being diluted back to 1 L with 10 mM TRIS pH 8.1, 3 M sodium chloride. This process was repeated a further two times, before concentrating back to 250 mL in preparation for purification.

#### Protein purification

All protein purification steps were carried out using a AKTA Pure Fast Protein Liquid Chromatography (FPLC) machine (Cytiva) following manufacturers guidelines. Proteins refolded via the standard refold protocol were dialysed into 10 mM TRIS and subjected to anion exchange purification using a POROS HQ50 column (Thermo Fisher Scientific). Protein was bound to the column using a flow rate of 20 mL min^-1^, before gradient elution with 10 mM TRIS, 1 M NaCl buffer at 5 mL min^-1^ in 1 mL fractions. Fractions containing the protein of interest, as determined by A_280_ were combined and concentrated to >1 mL using Amicon 10,000 kDa Millipore spin columns (Merck). Proteins refolded via the modified refold protocol were buffer exchanged into 10 mM TRIS, 3 M NaCl and subjected to Hydrophobicity Interaction Chromatography (HIC) purification using a HiTrap Capto Butyl Impress column (Cytiva). Protein was flowed through the HIC column at 5 mL/min and the flow through, which contained the protein of interest, was retained and concentrated to >1 mL using Amicon 10,000 kDa Millipore spin columns. Proteins purified via anion exchange or HIC were then purified by Size Exclusion Chromatography (SEC) using a Superdex 200/15 GL column (Cytiva). Proteins were injected down the column at 0.5 mL min^-1^ and eluted in 1 mL. The buffer used depended on the downstream experiments; PBS was used for use in tetramer staining and 10 mM TRIS, 10 mM NaCl buffer was used for crystallography. Fractions containing the protein if interest were retained for downstream experiments.

#### pMHC monomer biotinylation

After anion exchange, soluble pMHC protein containing a biotin tag was concentrated to 450 µL using Amicon 10,000 kDa Millipore spin columns. 1 µL of 1 mg mL^-1^ BirA enzyme (Avidity LLC, Auroro, CO, USA) and 50 µL of 10X SuperMix (Avidity) containing 55 mg/mL ATP (Avidity) were added to the soluble pMHC, that was then incubated overnight at room temperature. Once incubated, 15 mL of PBS buffer was added before concentrating to >1 mL in preparation for SEC.

#### Sitting-drop crystallization

TCR and pMHC protein crystals were grown at 18°C by sitting drop vapor diffusion using the following protocol. Directly after size exclusion chromatography TCR or peptide-MHC at a concentration of 10 mg mL^-1^, in a buffer of 10 mM Tris and 10 mM sodium chloride buffer, was dispensed in 200 nL drops into 3 well low profile Intelliplates (Art Robbins Instruments LLC, CA, USA) using the Gryphon liquid handling robot (Art Robins Instruments). Potential crystallization conditions were created by supplementing the protein with 200 nL from a 96 well crystallization screen and loading the reservoir with 60µL of the same condition. For crystallization of TCR:pMHC complexes, both components were mixed in a 1:1 molar ratio after size exclusion chromatography and followed the same process as described above. The PACT screen (Molecular Dimensions, Anatrace Products, LLC, OH, USA)^68^ and the T cell optimized crystal screen was used for this study^48^ with the conditions for each structure reported in the relevant statistics tables (Table S3). The plates were sealed using ClearVue adhesive film sheets (Molecular Dimensions) and stored at 18°C. Plates were periodically imaged using Rock Imager 2 (Formulatrix, MA, USA).

#### 3D structure determination, analysis and homology modeling

The reservoir solution of the successful crystallization condition was supplemented with 20% w/v ethyleneglycol (Molecular Dimensions). 1 µL of supplemented reservoir solution was added to the drop containing the crystals to provide cryoprotection. A magnetic acti-loop (Molecular Dimensions) was used to harvest the crystals from the drop, prior to storage in liquid nitrogen. The collected crystals were then sent to the Diamond Light Source synchrotron in Oxfordshire, UK. X-ray datasets were collected using a PILATUS 9M pixel detector at wavelength of 0.98 Å and consisted of 3600 images, with 0.1° oscillation and 0.1 s exposure at the I03, I04, and I04-1 MX beamlines at the Diamond Light Source. Datasets were processed using the DIALS, xia2, XDS, and AUTOPROC pipelines to produce reflection intensities and to determine unit cell dimensions and symmetry space group. The software used to process each structure is reported in the relevant statistics tables. The CCP4 version 7.1 software suit (Collaborative Computational Project No. 4, Didcot, UK) was used to derive 3D models from the reflection intensities.^69^ Phaser version 2.7 was used to conduct molecular replacement, producing a 3D structure solution.^70^ Win-Coot version 0.9.6 (Paul Emsley and Bernhard Lohkamp: https://strucbio.biologie.uni-konstanz.de/ccp4wiki/index.php/Coot) was used to adjust the amino acid sequence of the model to match the protein of interest, match the model to the electron density map and add relevant solvents to the 3D structure.^71^ REFMAC version 5.8 was used to refine the 3D structures.^72^ 3D protein structures were analysed using and images prepared using Pymol version 2.3.4 (Schrodinger, LLC). The reflection data and final coordinates were deposited in the PDB database www.rcsb.org (HLA A*02:01-NLSALGIFST PDB: 7Q98, MEL5:HLA A*02:01-NLSALGIFST PDB: 7Q99 MEL5:HLA A*02:01-LLLGIGILVL PDB: 7Q9A, MEL8:HLA A*02:01-EAAGIGILTV PDB: 7Q9B, HLA A*02:01-LLLGIGILV PDB: 7ZUC). For structures where we were unable to acquire structural data, homology modeling was used to provide a theoretical structure of the protein of interest. Homology modeling was conducted using the Modeller software suite.^73^ The MEL8:HLA A*02:01-EAAGIGILTV 3D structure was aligned with the MEL5:HLA A*02:01-LLLGIGILVL and MEL5:HLA A*0201-NLSALGIFST 3D structures respectively using PyMol to create models of the MEL8 TCR in complex with HLA A*02:01-LLLGIGILVL and MEL5:HLA A*02:01-NLSALGIFST. These models were aligned with their respective amino acid sequences using Modeller to form a theoretical 3D protein model which obeys known constraints of protein folding. The theoretical model was analysed as described above.

#### Data display

Unless stated otherwise, all data were displayed using GraphPad Prism software. TCR V-J usage plots were generated using VDJ tools.^74^

#### Cantigen

The CANTiGEN protein database is based on 1831 peptides with 87 HLAs, collated from the TANTIGEN platform (http://projects.met-hilab.org/tadb/), Cancer Research Institute peptide database (https://www.cancerresearch.org/en-us/scientists/meetings-and-resources/peptide-database) and manually curated peptides. The 1290 unique peptides contained 821 nonamers and 227 decamers; these were used to extract 1226 unique self proteins.^9^ containing 12,808,183 9-mer peptides and 12,770,552 10-mer peptides. CANTiGEN is freely accessible for academic use, through a user account (see https://cantigen.cf.ac.uk/loginform.php). The required inputs to the tool are a text file representing a matrix of PS-CPL values, one column for each of the 20 amino acids and one row for each position in the peptide, which are currently limited to a nonamer or decamer. Columns should be stacked, with one value per line and a total of either 180 or 200 values for 9-mer and 10-mer PS-CPL data respectively. The user can specify how many of the top peptides are required in the output. Optionally the user can enter a peptide sequence of interest, whose ranking in the output, if any, will be noted. Once completed, outputs can be downloaded. These consist of details of the top n peptides ranked by log likelihood score, and a heatmap representing the inputted PS-CPL matrix.

### Quantification and Statistical Analysis

For peptide sensitivity assays the EC_50_ of activation was calculated using GraphPad Prism. Bar graphs of tetramer staining, T cell activation or killing display single values, or the mean of duplicates of the same condition, with individual data points displayed. Error bars depicting SEM are displayed when triplicate conditions were performed.

### Additional Resources

TILs and autologous melanoma cells were provided from patients MM909.11, MM909.15 and MM909.24 enrolled on clinical trial (NCT00937625) entitled T cell Based Immunotherapy for Melanoma (https://clinicaltrials.gov/ct2/show/NCT00937625).

## Supplementary Material

**Supplemental Information**

Supplemental information can be found online at https://doi.org/10.1016/j.cell.2023.06.020.

Supplementary Table S1

Supplementary Table S2

Supplementary Table S3

Supplementary Table S4

Supplementary Table S5

Supplemental figures

## Data Availability

Patient-related data, including RNA sequencing, not included in the paper may be subject to patient confidentiality. Any data that can be shared will be released via material transfer agreement and data processing agreements. RNAseq data for other cancer cells was sourced from TRON Cell Line Portal (http://celllines.tron-mainz.de/). TCR sequencing data is available from: https://vdjdb.cdr3.net/. All crystal datasets have been deposited in the Protein Database: https://www.rcsb.org Accession numbers are listed in the key resources table. All original code has been deposited at GitHub and is publicly available as of the date of publication. The DOI is listed in the key resources table. Any additional information required to reanalyze the data reported in this paper is available from the lead contact upon request.

## In brief

Detailed characterization of the recognition and activation characteristics of T cells from successful therapy against melanoma unveils that individual T cells recognize multiple tumor-associated antigens simultaneously; elicitation or engineering of such “multipronged” T cells may be an effective means of enhancing the efficacy of T cell cancer therapy.

## Highlights

- Individual T cells from successful immunotherapy recognize multiple cancer types
- A deciphering pipeline identifies new epitopes recognized by these “orphan” T cells
- Single T cells recognize multiple, different tumor-associated antigens simultaneously
- “Multipronged” TCRs exhibit superior cancer recognition compared with monospecific TCRs
