# Supplementary material for: Targeting of multiple tumor-associated antigens by individual T cell receptors during successful cancer immunotherapy
Source: Cell. Author manuscript; Available in PMC 2026 Oct 24. (PMC7619633; doi:10.1016/j.cell.2023.06.020)
Supplement: Supplementary file 1 [file EMS220778-supplement-Supplemental_figures.pdf]

Supplemental figures

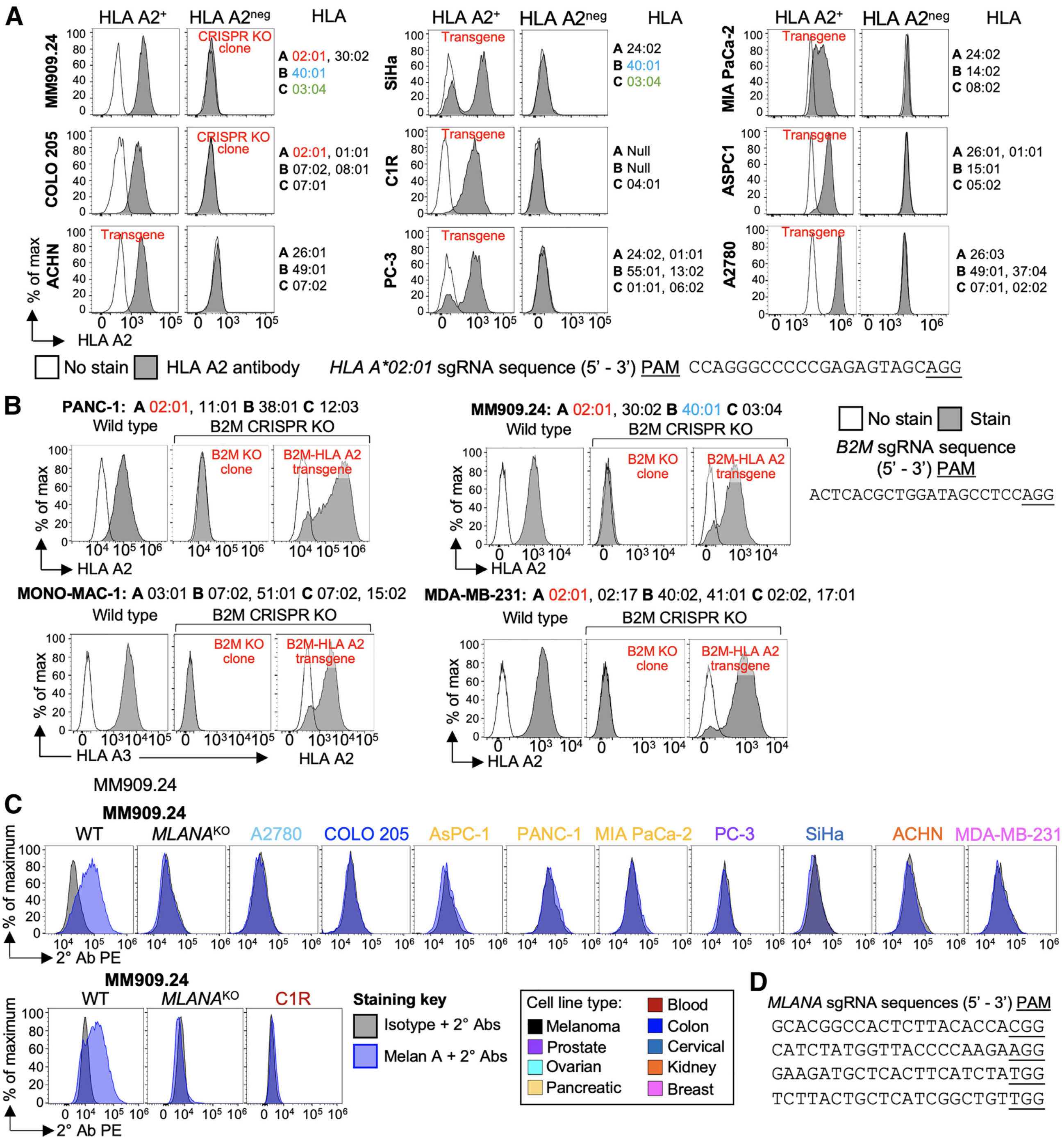

Figure S1. Melan A staining of non-melanoma cells and genetic modifications of various cancer cell lines, related to Figures 1 and 4

(A) *HLA A*02:01*⁺ cancer cell lines MM909.24 and COLO 205, were transduced with Cas9 and a single guide (sg)RNA targeting *HLA A*02:01*, whereas naturally *HLA A*02:01*^{neg} cancer cell lines ACHN, SiHa, C1R, PC-3, Mia PaCa-2, A2780, and AsPC1 were transduced with *HLA A*02:01*. Staining with an HLA A*02 antibody is shown. HLA type of each tumor line is indicated, with matching HLAs to MM909.24 shown in color.

(B) To reduce alloreactivity toward cancer cell lines all HLA class I was removed from the cell surface, then HLA A*02:01 expressed or re-introduced: CRISPR-Cas9 beta-2 microglobulin (B2M) KO clones of cancer cell lines expressed as a single chain *B2M-HLA A*02:01* transgene. B2M KO assessed by B2M antibody

(legend continued on next page)

staining (not shown) and HLA A*02 (PANC-1, MM909.24, and MDA-MB-231) or HLA A*03 (MONO-MAC-1) antibody staining. The MM909.24 ± HLA A*02:01 were generated by methods described in (A) and (B); the melanoma B2M KO and B2M-HLA A*02:01 were only used in Figure 1L, whereas the Cas9 mediated KO cells were used elsewhere in the study.

(C) Melanoma MM909.24 was transduced with 4 individual sgRNAs targeting *MLANA* (shown in D), the resulting knockout (KO) lines cloned, then clones screened by intracellular staining for Melan A using a primary unconjugated antibody and PE conjugated secondary antibody with an isotype antibody used as a negative control, according to the key. MM909.24 clones were mixed to create a KO line, which did not stain with Melan A antibody. Within the same experiments (upper and lower panels are from two different experiments), intracellular staining for Melan A of non-melanoma cancer cell lines was also performed.

(D) sgRNAs sequences used to ablate Melan A expression in melanoma MM909.24.

CellPress
OPEN ACCESS

A

MEL8α native sequence: leader, TRAV12-2*02, CDR3α, TRAJ8*01 and TRAC1 (splice site):

atgaaatccttgagagttttactagtgatcctgtggcttcagttgagctgggtttggagccaacagaaggaggtgg
agcagaattctggacccctcagtgttccagagggagccattgcctctctcaactgcacttacagtgaccgaggttc
ccagtccttcttctggtacagacaatattctgggaaaagccctgagttgataatgtccatatactccaatggtgac
aaagaagatggaaggtttacagcacagctcaataaagccagccagtatgtttctctgctcatcagagactcccagc
ccagtgattcagccacctacctctgtgccgtgcagaaacttgtatttggaactggcacccgacttctggtcagtcc
aaatatccagaaccctgaccctgccgtgtaccagctgagagactctaaatccagtgacaagtctgtctgcctattc
accgattttgattctcaaacaaatgtgtcacaaagtaaggattctgatgtgtatatcacagacaaaactgtgctag
acatgaggtctatggacttcaagagcaacagtgctgtggcctggagcaacaaatctgactttgcatgtgcaaacgc
cttcaacaacagcattattccagaagacaccttcttccccagcccagaaagttcctgtgatgtcaagctggtcgag
aaaagctttgaaacagatacgaacctaaactttcaaaacctgtcagtgattgggttccgaatcctcctcctgaaag
tggccgggtttaatctgctcatgacgctgcggctgtggtccagc

MEL8β native sequence: leader, TRBV6-5*01, CDR3β, TRBD2*02 TRBJ2-7*01 and TRBC2*01 (splice site):

atgagcatcggcctcctgtgctgtgcagccttgtctctcctgtgggcaggtccagtgaatgctggtgtcactcaga
ccccaaaattccaggtcctgaagacaggacagagcatgacactgcagtgtgcccaggatatgaaccatgaatacat
gtcctggtatcgacaagacccaggcatggggctgaggctgattcattactcagttggtgctggtatcactgaccaa
ggagaagtccccaatggctacaatgtctccagatcaaccacagaggatttcccgctcaggctgctgtcggctgctc
cctcccagacatctgtgtacttctgtgccagcagttactcctttactgaggcgacctacgagcagtacttcgggcc
gggcaccaggctcacggtcacagaggacctgaaaaacgtgttcccacccgaggtcgctgtgtttgagccatcagaa
gcagagatctcccacacccaaaaggccacactggtgtgcctggccacaggcttctaccccgaccacgtggagctga
gctggtgggtgaatgggaaggaggtgcacagtggggtcagcacagacccgcagcccctcaaggagcagcccgccct
caatgactccagatactgcctgagcagccgcctgagggtctcggccaccttctggcagaacccccgcaaccacttc
cgctgtcaagtccagttctacgggctctcggagaatgacgagtggacccaggatagggccaaacctgtcacccaga
tcgtcagcgccgaggcctggggtagagcagactgtggcttcacctccgagtcttaccagcaaggggtcctgtctgc
caccatcctctatgagatcttgctagggaaggccaccttgtatgccgtgctggtcagtgccctcgtgctgatggcc
atggtcaagagaaaggattccagaggc

B

MEL8α protein construct: V-domain, CDR1α, CDR2α, CDR3α, splice site amino acid, C-domain, non-native cysteine and stop codons (**):

MQKEVEQNSGPLSVPEGAIASLNCTYSDRGSQSFFWYRQYSGKSPELIMSIYSNGDKEDGRFTAQLN
KASQYVSLLIRDSQPSDSATYLCAVQKLVFGTGTRLLVSPNIQNPDPAVYQLRDSKSSDKSVCLFTD
FDSQTNVSQSKDSDVYITDKCVLDMRSMDFKSNSAVAWSNKSDFACANAFNNSIIPEDTFFPSPESS
**

MEL8β protein construct: V-domain, CDR1β, CDR2β, CDR3β, splice site amino acid, C-domain, non-native cysteine and stop codons (**):

MNAGVTQTPKFQVLKTGQSMTLQCAQDMNHEYMSWYRQDPGMGLRLIHYSVGAGITDQGEVPNGYNV
SRSTTEDFPLRLLSAAPSQTSVYFCASSYSFTEATYEQYFGPGTRLTVTEDLKNVFPPEVAVFEPSE
AEISHTQKATLVCLATGFYPDHVELSWWVNGKEVHSGVCTDPQPLKEQPALNDSRYALSSRLRVSAT
FWQDPRNHFRCQVQFYGLSENDEWTQDRAKPVTQIVSAEAWGRAD**

Figure S2. Sequence of the MEL8 TCR, related to Figures 3 and S6

(A) Native nucleotide sequence of TCRα and TCRβ genes showing V(D)J assignment.

(B) Bacterially expressed protein sequences manufactured in *E. coli* and refolded to make soluble MEL8 TCR for biophysical and structural studies. Sequences include non-native cysteine residues as indicated in bold underlined red text to form non-native disulphide bonding between the TCRα and TCRβ constant domains. Two other substitutions in the TCRβ chain that aid refolding are indicated in bold text. The Cys to Ala substitution was included to remove the possibility of incorrect disulphide bind formation.

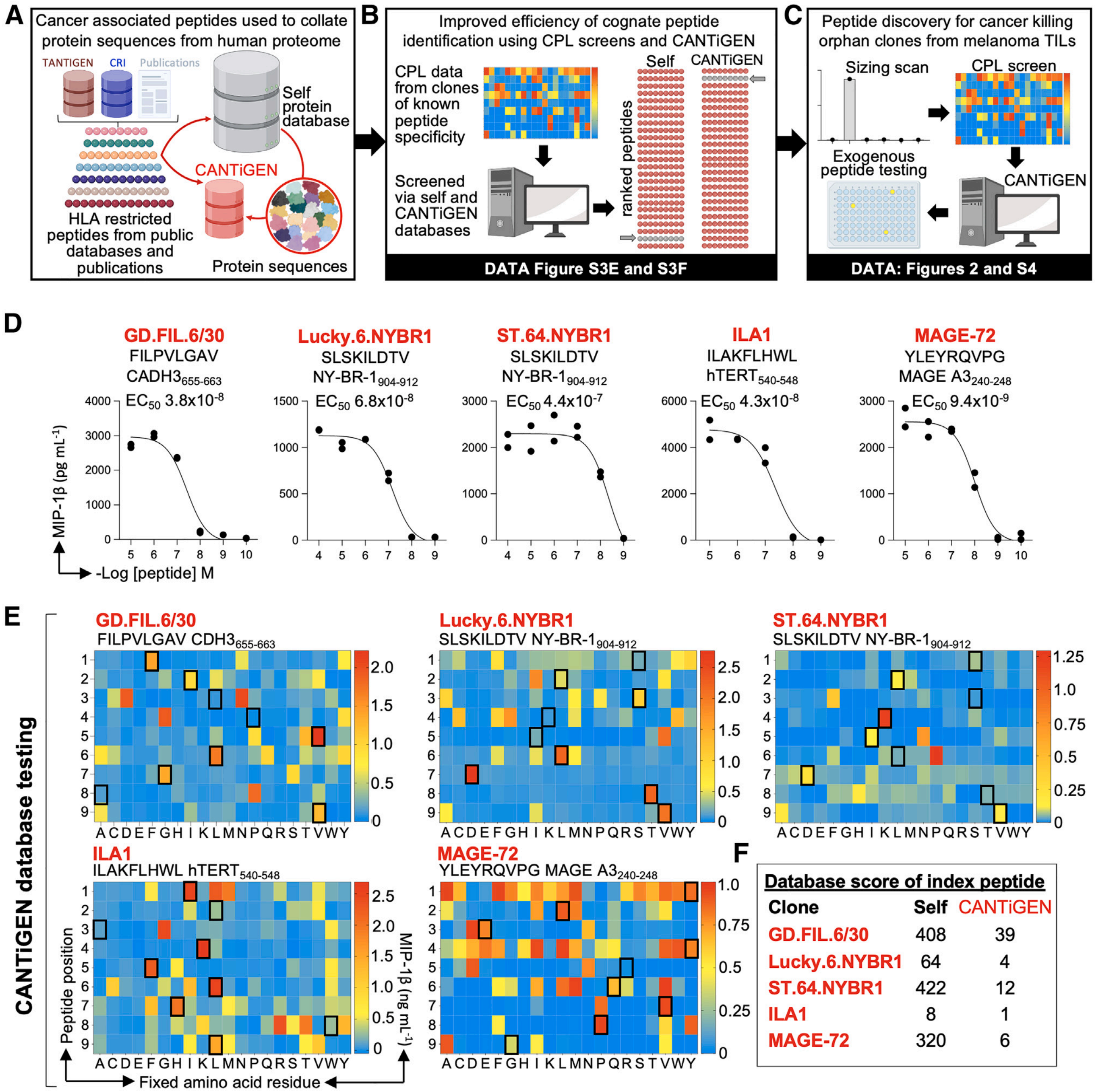

Figure S3. Efficient identification of TAA-derived peptides by PS-CPL screening and interrogation of a cancer-associated proteomics database, related to Figure 2

(A) HLA-restricted cancer-associated epitopes were used to assemble the CANTiGEN database consisting of cancer-associated proteins and described epitopes.

(B) Positional scanning combinatorial peptide library (PS-CPL) data of clones with known peptide specificity was used to screen the human self-protein and CANTiGEN databases.

(C) T cell clones or TCR-T cells undergo peptide size scanning, matched-length PS-CPL screening then testing with candidate peptides identified by CANTiGEN.

(D) Peptide recognition by five HLA*02:01-restricted T cell clones generated using our T cell libraries technique[50] and other methods that recognize known sequences.[21,75,76] Clone name (bold red text), index peptide and the protein source of the peptide and residue numbers are shown above the graph. EC_{50} values of activation are displayed.

(E) Testing the efficiency of CANTiGEN versus human self-protein database screening using PS-CPL data generated using the five CD8 T cell clones shown in (D). Clone name, peptide sequence, and protein origin (peptide residues shown in subscript) displayed above each heatmap of the PS-CPL data. Index peptide sequence for each clone is highlighted with a black box on the heatmap.

(F) Ranking of index peptide of each clone predicted by the human-self and CANTiGEN databases. CANTiGEN substantially improves the ranking of the index peptide compared with results generated searching the human proteome (self) database.

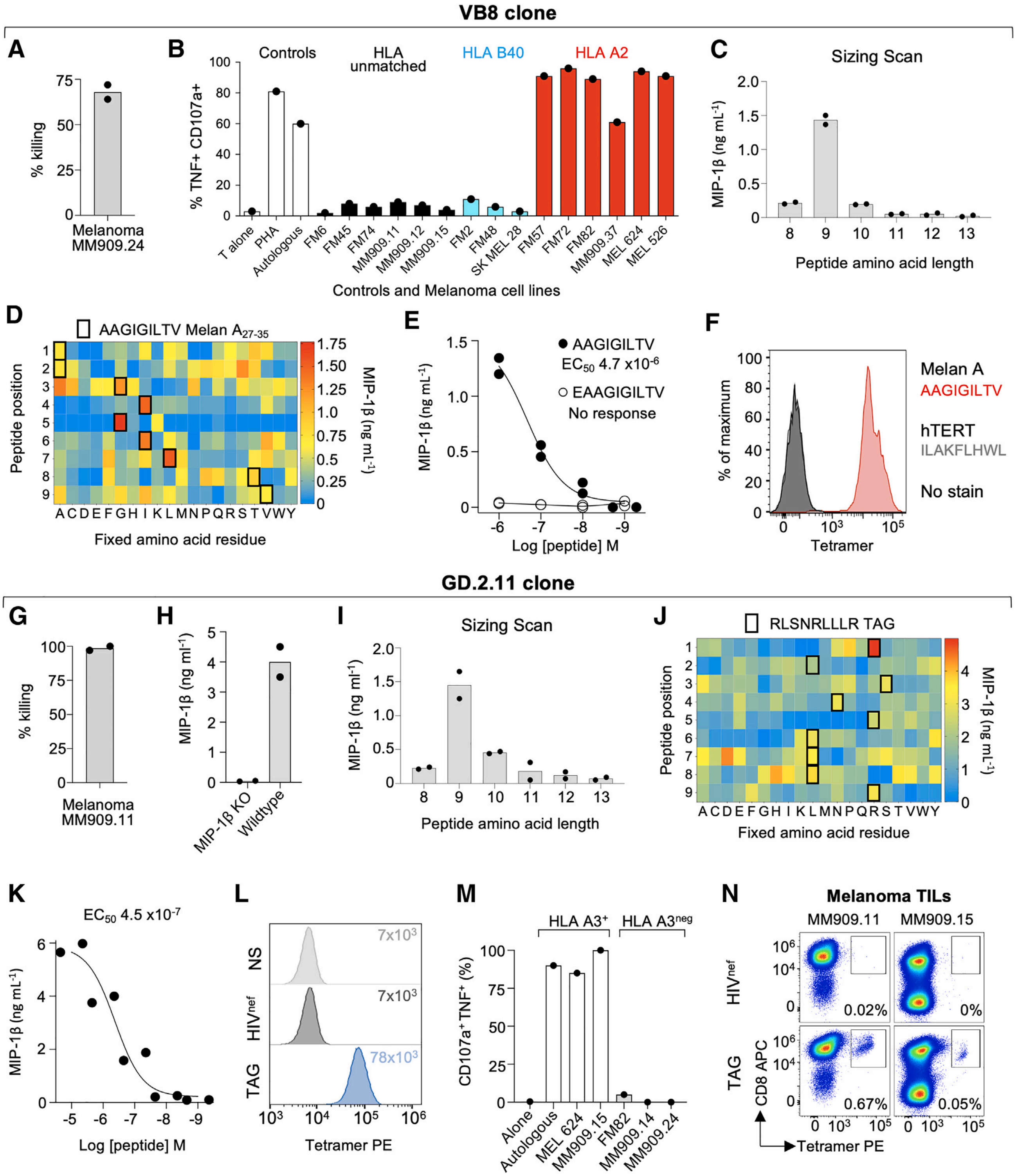

Figure S4. Screening of CANTiGEN for peptide discovery of orphan clones, related to Figure 2

(A–F) Data generated using orphan T cell clone HLA A*02:01 restricted clone VB8 of unknown peptide reactivity, from patient MM909.24 TIL.

(A) VB8 kills the autologous melanoma line in cytometry based killing assay.

(B) VB8 recognizes the autologous and HLA A*02^{+} melanoma cell lines but not HLA unmatched lines or those matched at HLA B*40, which is also expressed by patient MM909.24.

(C) VB8 shows a preference for 9-mer peptides in overnight MIP-1β ELISA.

(legend continued on next page)

(D) 9-mer PS-CPL data from VB8 displayed as a heatmap. CANTiGEN predicted the 9-mer AAGIGILTV (Melan A_{27-35}) as the most likely antigen using data shown in heatmap.

(E) Peptide titration confirmed recognition of AAGIGILTV peptide, but not the 10-mer EAAGIGILTV (Melan A_{26-35}).

(F) VB8 stains with HLA A*02:01-AAGIGILTV tetramer.

(G–M) Data using orphan T cell clone GD.2.11 of unknown peptide reactivity and HLA restriction, from the TIL used to induce complete remission in patient MM909.11.

(G) GD.2.11 kills the autologous melanoma line in cytometry based killing assay.

(H) Generation of autologous lymphoblastoid cell line (LCL) to act as antigen presenting cells (APCs) expressing all HLA from patient MM909.11. MIP-1β knockout from the LCL so not occlude T cells assays using MIP-1β as the output.

(I) GD.2.11 shows a preference for 9-mer peptides in overnight MIP-1β ELISA using autologous MIP-1 β knockout LCL as APCs.

(J) 9-mer PS-CPL data displayed as a heatmap, generated with GD.2.11 using MIP-1β knockout LCL as APC. HLA A*03 restricted peptide RLSNRLLLR from TAG (tumor-associated gene) was the top hit predicted by CANTiGEN.

(K) Recognition of RLSNRLLLR peptide titration (MIP-1β knockout LCL as APC).

(L) T107 assay (5 h) of CD8 T cell clone GD.2.11 HLA A*03:01-matched or mismatched melanoma cell lines.

(M) GD.2.11 stains with HLA A*03:01-RLSNRLLLR tetramer confirmed HLA restriction and peptide recognition. Mean fluorescence intensities are shown for each stain. Irrelevant HLA A*03:01 tetramer made with from HIV-Nef_{73-82} (QVPLRPMTYK).

(N) HLA A*03:01-RLSNRLLLR tetramer staining of TIL (passage-2) from HLA A*03:01^{+} melanoma patients MM909.11 and MM909.15. Irrelevant tetramer as in (F).

CellPress
OPEN ACCESS

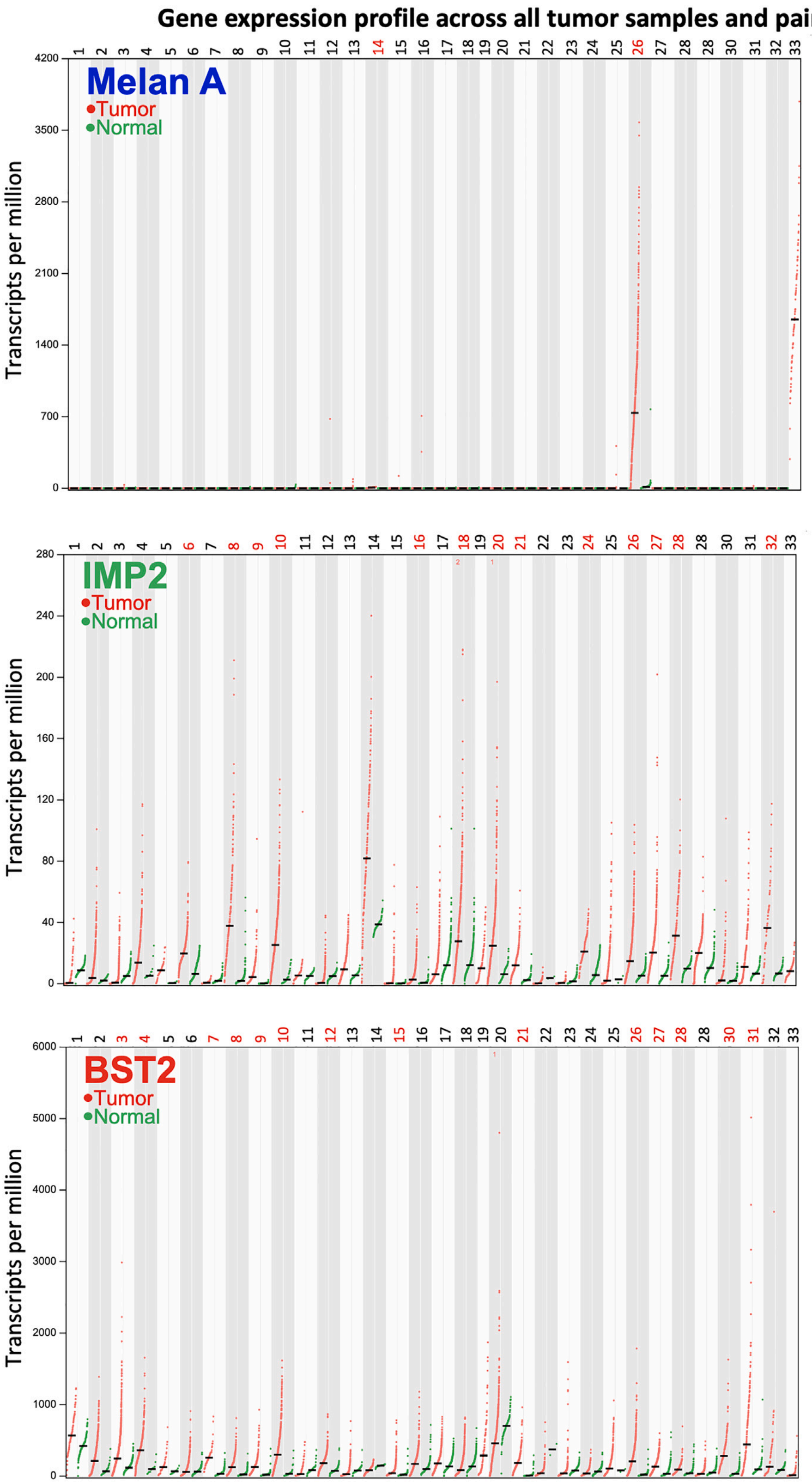

1. Adrenocortical carcinoma
2. Bladder Urothelial Carcinoma
3. Breast invasive carcinoma
4. Cervical squamous cell carcinoma and endocervical adenocarcinoma
5. Cholangiocarcinoma
6. Colon adenocarcinoma
7. Diffuse large B cell lymphoma
8. Esophageal carcinoma
9. Glioblastoma multiforme
10. Head and Neck squamous cell carcinoma
11. Kidney Chromophobe
12. Kidney renal clear cell carcinoma
13. Kidney renal papillary cell carcinoma
14. Acute Myeloid Leukemia
15. Low-grade gliomas
16. Liver hepatocellular carcinoma
17. Lung adenocarcinoma
18. Lung squamous cell carcinoma
19. Mesothelioma*
20. Ovarian serous cystadenocarcinoma
21. Pancreatic adenocarcinoma
22. Pheochromocytoma and Paraganglioma
23. Prostate adenocarcinoma
24. Rectum adenocarcinoma
25. Sarcoma
26. Skin Cutaneous Melanoma
27. Stomach adenocarcinoma
28. Testicular Germ Cell Tumors
29. Thyroid carcinoma
30. Thymoma
31. Uterine Corpus Endometrial Carcinoma
32. Uterine Carcinosarcoma
33. Uveal Melanoma*

*data displayed for tumor tissue only

(legend on next page)

Figure S5. Melan A, BST2, and IMP2 expression in healthy versus cancer tissue, related to Figure 2
Melan A, IMP2, and BST2 mRNA expression in paired cancer and healthy tissue samples for 33 cancer types. Figures from gene expression profiling interactive analysis (GEPIA) web server.[77] Melan A protein is present at low levels in melanocytes but overexpressed by most melanomas. IMP2 protein has only been found in human gametes. It is unclear how mRNA levels correlate with BST2 protein expression. Data for tumor but not healthy tissue are displayed for mesothelioma (19) and uveal melanoma (33). Red numbers indicate number of samples that are off the scale of the graphs as drawn (see GEPIA web server; http://gepia.cancer-pku.cn).

CellPress
OPEN ACCESS

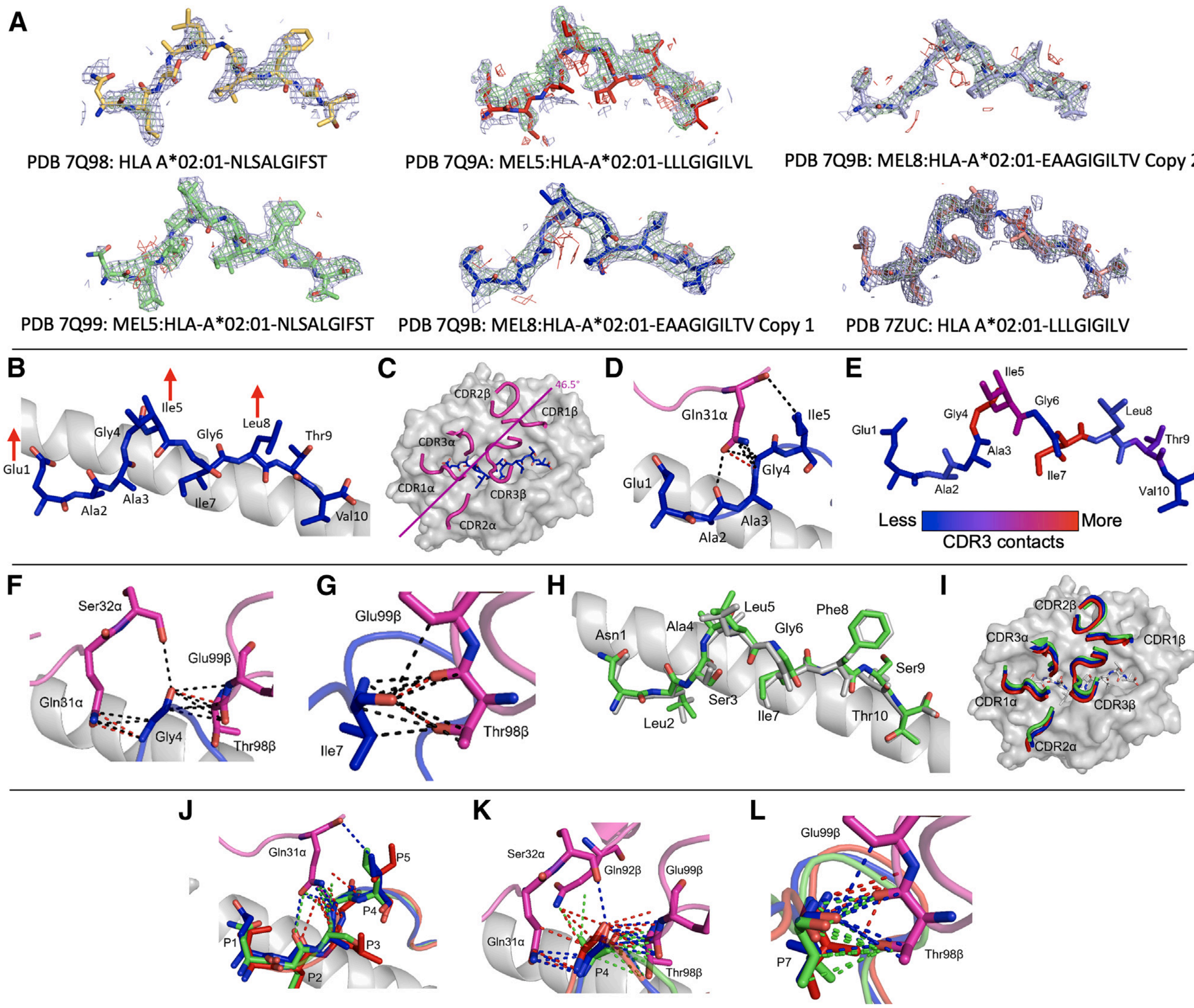

Figure S6. Structural dissection of multipronged TCRs and epitopes, related to Figure 3

(A) Final coordinates of the peptides of each structure in an omit map after solving each structure with Phaser using a model not including the peptide. The density is displayed in light blue from the observed map, in green contours from the positive difference map, and in red for the negative contours from the difference map.

(B) Melan $A_{26–35}$ (EAAGIGILTV) peptide (blue) when bound to MEL8 shown as sticks. MHC alpha helix (gray) shown as cartoon for orientation. Red arrows highlight amino acid protrusion.

(C) Top-down view of Melan A peptide (blue sticks) presented by HLA A*02:01 (gray, shown as surface). MEL8complementarity determining region (CDR) loops (magenta) are shown as cartoon. Crossing angle is indicated by the magenta line.

(D) Close up of MEL8 Gln31α (magenta) interacting with the Melan A peptide (blue). van der Waals forces (black dotted lines) and hydrogen bonds (red dotted lines) are shown.

(E) Heatmap of Melan A peptide showing number of interactions each residue makes with MEL8 CDR loops.

(F and G) Interactions between MEL8 (magenta sticks) and Gly4 (F) and Ile7 (G) of Melan A peptide (blue). van der Waals forces (black dotted line) and hydrogen bonds (red dotted lines) are shown.

(H) Presentation of the $IMP2_{367–376}$ NLSALGIFST peptide presented by HLA A*02:01 when unbound (white) and bound to the MEL5 TCR (green).

(I) Top-down view showing MEL8 CDR loops in complex Melan A (blue), $BST2_{22–31}$ (LLLGIGILVL, red), and IMP2 (green) peptides. Representative peptide shown in white.

(J) Close up of MEL8 Gln31α (magenta) interacting with the Melan A, BST2, and IMP-2 peptides.

(K and L) MEL8 (magenta) residues which interact with P4 (K) and P7 (L) of Melan A (blue), BST2 (red), and IMP-2 (green) peptides, respectively. Bonds shown in Figures S6J–S6L involve the Melan A (blue), BST2 (red), and IMP-2 (green) peptides, respectively.

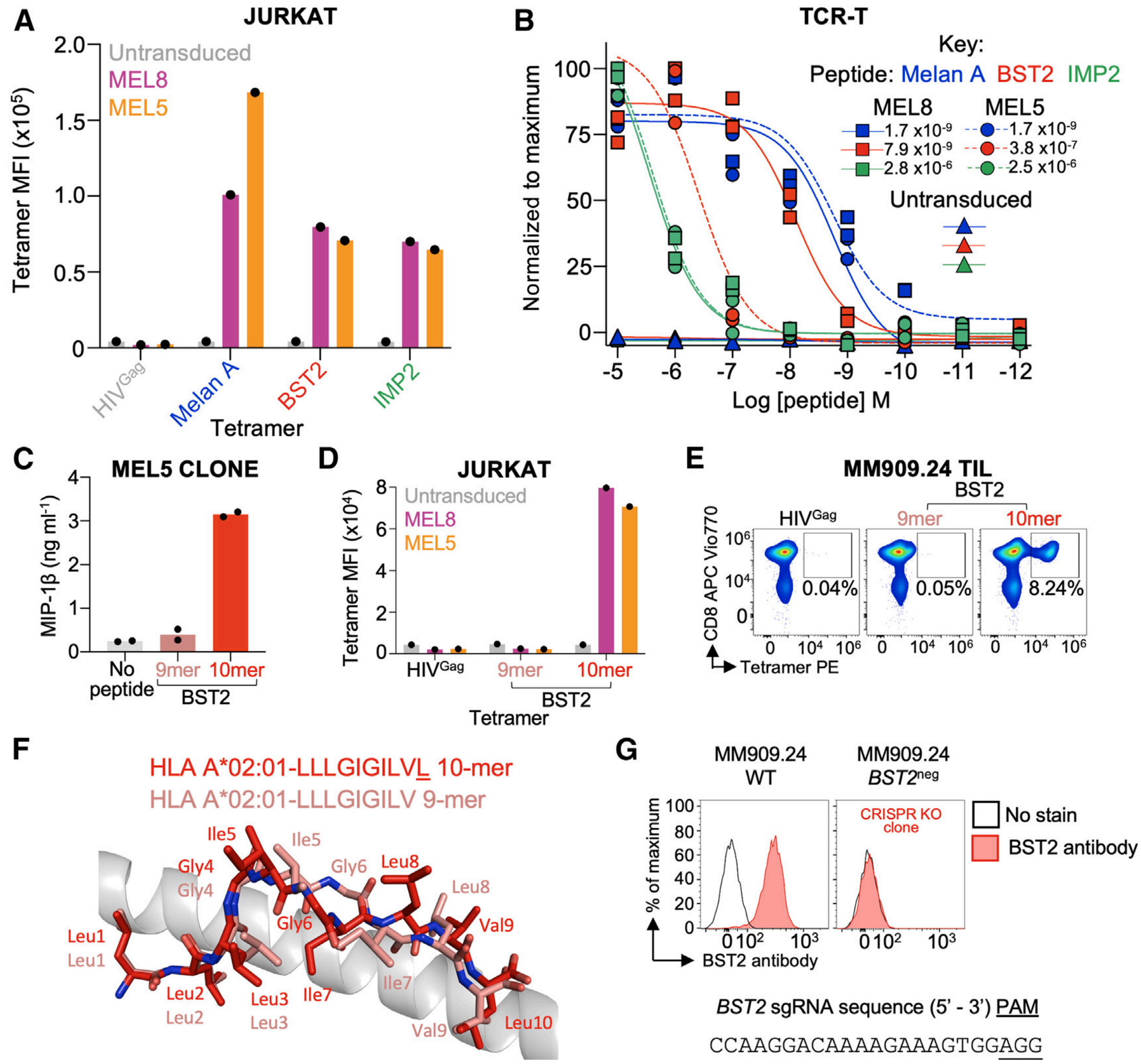

Figure S7. Ligand recognition by MEL8 and MEL5 TCRs, and lack of recognition of multipronged TCRs toward the 9-mer version of the BST2 peptide, related to Figures 2 and 3

(A and B) Comparison of the MEL8 and MEL5 TCRs: (A) $CD8^+$ Jurkat cells transduced with MEL8 or MEL5 TCRs with a rCD2 co-marker. Stained with Melan A_{26-35} (EAAGIGILTV, Melan A_{10}), $BST2_{22-31}$ (LLLGIGILVL, $BST2_{10}$), $IMP2_{367-376}$ (NLSALGIFST, $IMP2_{10}$), and irrelevant (HIV Gag_{77-85} SLYNTVATL) tetramers. Gated on viable $rCD2^+$ cells. See Table S1 for peptide details.

(B) Primary CD8 T cells from the same donor transduced with MEL8 and MEL5 TCRs and tested in overnight titration assays with Melan A, BST2, and IMP2 peptides, followed by MIP-1β ELISA. Reactivity normalized to maximum activation for each titration set. Untransduced normalized to maximum activation for either MEL8 or MEL5 for the corresponding peptide. EC_{50} values are displayed in the key.

(C–F) Functional and structural focus on the BST2 9-mer and 10-mer peptides. In a previous study, a shorter 9-mer BST2 peptide lacking the C terminus leucine (LLLGIGILV resiudes 22-30, $BST2_9$) was predicted by SYFPEITHI and BIMASS software to bind to HLA A*02:01 and used to generate myeloma-specific T cells.[45]

(C) In keeping with its preference for peptides of 10 amino acids in length, the MEL5 clone reacted poorly toward the $BST2_9$ peptide, in comparison with the $BST2_{10}$ peptide in overnight activation assay by MIP-1β ELISA.

(D) $CD8^+$ Jurkat cells transduced with MEL8 or MEL5 TCRs with a rCD2 co-marker. Stained with $BST2_9$, $BST2_{10}$, and irrelevant (HIV Gag) tetramers. Gated on viable $rCD2^+$ cells. The $BST2_{10}$ and irrelevant tetramer data are the same that appears in (A).

(E) TIL (passage 3) from melanoma patient MM909.24 stained with $BST2_9$, $BST2_{10}$, and irrelevant (HIV Gag) tetramers. Gated on viable $CD3^+$ T cells.

(F) Presentation of the $BST2_9$, which diffracted to 1.89 Å (pink; PBD: 7ZUC; Table S4) and the $BST2_{10}$ (red) peptides by HLA A*02:01 (gray cartoon). The structural differences between the $BST2_9$ and $BST2_{10}$ peptides are likely due to the HLA accommodating the differing peptide lengths within the same space, as the location of the N- and C-terminal anchor residues are fixed as position 2 and the C-terminal residue, respectively. We hypothesize that this difference in peptide presentation is responsible for the lack of response toward the $BST2_9$ peptide by MM909.24 TIL, MEL5, and MEL8 compared with $BST2_{10}$ peptide.

(G) BST2 knockout from melanoma MM909.24; staining of wild-type line and knockout clone and sequence of the sgRNA used.

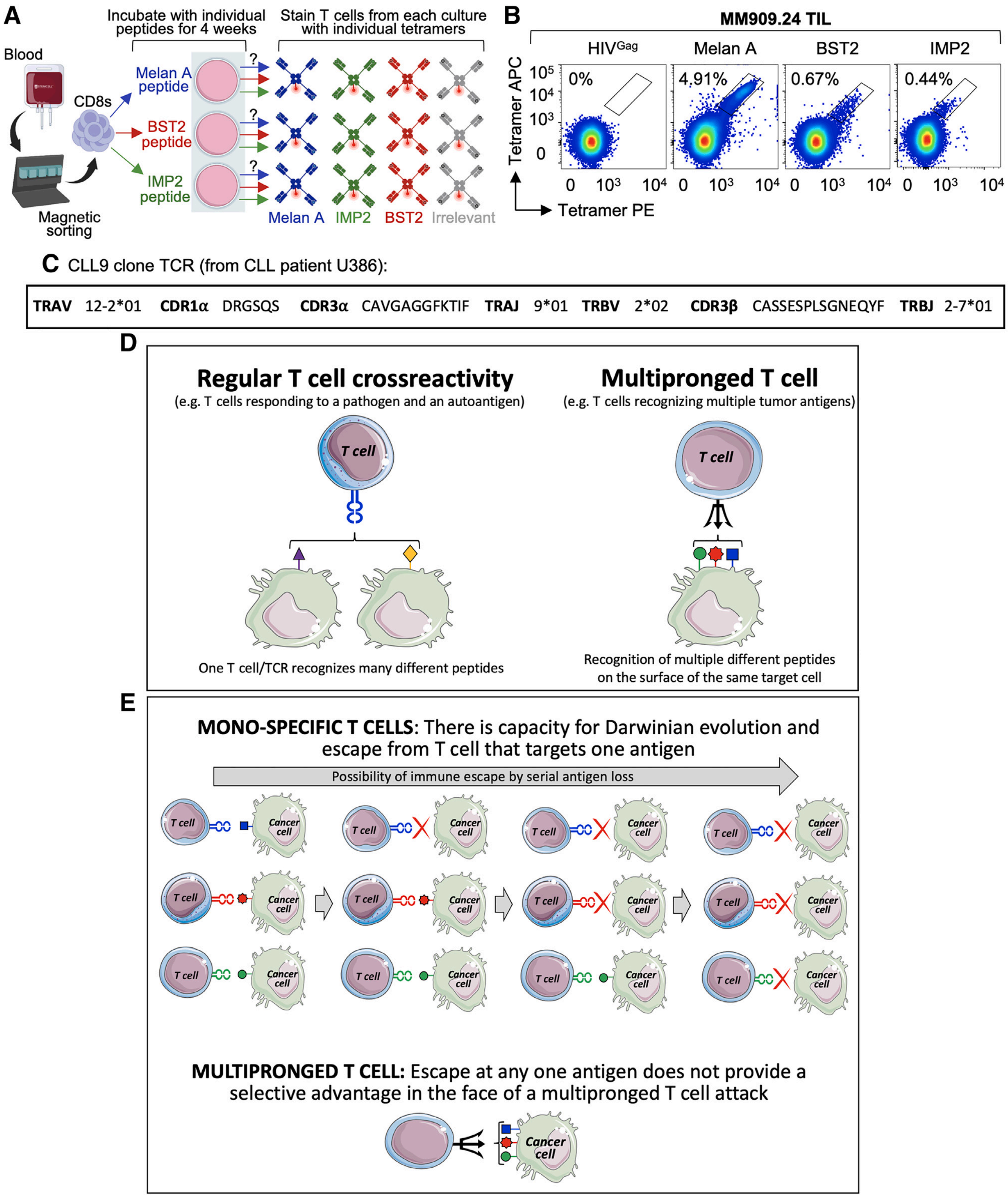

Figure S8. Assay setup for peptide priming of healthy donor T cells, tetramer sorting from TIL MM909.24 for TCR clonotyping and summary of the potential benefits of multipronged T cells, related to Figures 5 and 6

(A) CD8 T cells from a healthy donor were split over three cultures and incubated with either Melan $A_{26–35}$ (EAAGIGILTV), $BST2_{22–31}$ (LLLGIGILVL), or $IMP2_{367–376}$ (NLSALGIFST) peptide, each at 25 μM. Subsequently, each culture was stained with Melan A, BST2, IMP2, or irrelevant tetramers to assess the specificities of the T cells induced by each of the peptides.

(legend continued on next page)

(B) Staining of TIL (passage-1) from melanoma patient MM909.24 using HLA A*02:01 tetramers for sorting by flow cytometry and sequencing of the TCRs. Samples were co-stained with APC and PE tetramers assembled with the same epitope. HLA A*02:01-SLYNTVATL (HIV Gag_{77-85}) used as an irrelevant tetramer.[20] The percentage of $tetramer^{+}$ T cells is shown.

(C) TCR details of the CLL9 clone grown from CLL patient U386.

(D) Recognition of multiple epitopes on the same cell distinguishes multipronged T cell recognition from regular T cell crossreactivity such as during molecular mimicry-induced triggering of autoimmune disease where a T cell responds to different epitopes on different target cells.

(E) There are many potential advantages to multipronged T cell recognition in addition to the fact that such T cells can respond more potently as they “see” a higher density of antigen at the target cell surface. For instance, it would be more difficult to escape from a multipronged T cell by loss of peptide antigen.
